# UV/Vis Spectroscopy of Copper Formate Clusters: Insight into Metal‐Ligand Photochemistry

**DOI:** 10.1002/chem.202000280

**Published:** 2020-06-18

**Authors:** Tobias F. Pascher, Milan Ončák, Christian van der Linde, Martin K. Beyer

**Affiliations:** ^1^ Institut für Ionenphysik und Angewandte Physik Universität Innsbruck Technikerstraße 25 6020 Innsbruck Austria

**Keywords:** cluster compounds, copper, density functional calculations, photochemistry, UV/Vis spectroscopy

## Abstract

The electronic structure and photochemistry of copper formate clusters, Cu^I^_2_(HCO_2_)_3_^−^ and Cu^II^_*n*_(HCO_2_)_2*n*+1_^−^, *n*≤8, are investigated in the gas phase by using UV/Vis spectroscopy in combination with quantum chemical calculations. A clear difference in the spectra of clusters with Cu^I^ and Cu^II^ copper ions is observed. For the Cu^I^ species, transitions between copper d and s/p orbitals are recorded. For stoichiometric Cu^II^ formate clusters, the spectra are dominated by copper d–d transitions and charge‐transfer excitations from formate to the vacant copper d orbital. Calculations reveal the existence of several energetically low‐lying isomers, and the energetic position of the electronic transitions depends strongly on the specific isomer. The oxidation state of the copper centers governs the photochemistry. In Cu^II^(HCO_2_)_3_^−^, fast internal conversion into the electronic ground state is observed, leading to statistical dissociation; for charge‐transfer excitations, specific excited‐state reaction channels are observed in addition, such as formyloxyl radical loss. In Cu^I^_2_(HCO_2_)_3_^−^, the system relaxes to a local minimum on an excited‐state potential‐energy surface and might undergo fluorescence or reach a conical intersection to the ground state; in both cases, this provides substantial energy for statistical decomposition. Alternatively, a Cu^II^(HCO_2_)_3_Cu^0−^ biradical structure is formed in the excited state, which gives rise to the photochemical loss of a neutral copper atom.

## Graphical abstract

**Oxidation state is key**: Copper formate clusters in oxidation states +I and +II in the gas phase are investigated by using a combination of UV/Vis spectroscopy and quantum chemical calculations. The electronic transitions and photochemistry are governed by the oxidation state. Whereas ligand‐to‐metal charge‐transfer states in Cu^II^ can lead to photochemical formyloxyl radical loss, ejection of a neutral copper atom is observed in the Cu^I^ species.

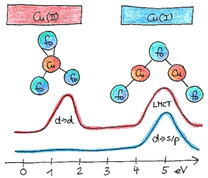

## Introduction

With global warming becoming an ever‐increasing problem, efficient activation and transformation of carbon dioxide becomes a desirable option for carbon capture and usage (CCU). Transformation into formic acid and methanol are very attractive candidates.^1^, ^2^, ^3^ Formic acid is regarded as the simplest stable form of activated carbon dioxide and is widely investigated.^4^, ^5^, ^6^, ^7^ In these transformations, formate is considered as a key intermediate for the hydrogenation of carbon dioxide.^2^, ^4^ Furthermore, the highly selective decomposition of formic acid could play a key role in hydrogen storage applications.^8^, ^9^ Copper‐based catalysts are highly active and applied as a carboxylation agent.^10^, ^11^ Additionally, they are already widely researched and used in industry within methanol synthesis.^12^, ^13^, ^14^, ^15^ However, the nature of the active sites of catalytic materials and the respective electronic configuration remain hotly debated.^16^, ^17^

For a mechanistic understanding, gas‐phase investigations of well‐defined, size‐selected model systems, in combination with quantum chemical calculations, are useful.^18^, ^19^, ^20^ Additionally, gas‐phase approaches can be translated through size‐selected cluster deposition to study surface effects within heterogeneous catalysis.^21^, ^22^ In the gas phase, it was shown that the activation of methane to methanol might take place on a CuO^+^ center.^23^ Al_2_CuO_5_^+^ complexes have been found to selectively convert methane into CH_2_O.^24^, ^25^ Furthermore, it was revealed that hydrated doubly charged copper ions, Cu^2+^(H_2_O)_*n*_, underwent charge separation until a critical size of *n=*8.^26^, ^27^, ^28^ Additionally, gas‐phase experiments and quantum chemical calculations have been applied successfully to understand the mechanisms behind the activation of carbon dioxide on copper hydrides,^29^, ^30^, ^31^, ^32^, ^33^ with copper formate clusters as particularly suitable model systems.^31^, ^33^

Recently, we showed that the formation of formic acid within copper formate clusters took place in the gas phase in a redox reaction through proton‐coupled electron transfer for clusters with two copper centers, whereas it proceeded through hydrogen atom transfer with a single copper center.^31^ Furthermore, we observed that the size of the cluster and the copper oxidation state of +II played a key role in the reaction of formate towards formic acid, emphasizing the importance of electronic structures for catalysis. Larger Cu^II^ formate anions have been found to fragment in a cascade towards clusters with one or two copper centers, upon which redox reactions can be observed, followed by decarboxylation towards Cu^I^ hydrides as the predominant process.^33^ The formate anion in various environments has been investigated, in detail, by means of infrared spectroscopy and theory due to anomalies within the vibrational spectrum with intense anharmonicities,^34^, ^35^, ^36^, ^37^, ^38^ and its electronic structure has been studied in solution.^39^ In the catalytically relevant case of formate ligated copper, structural data on copper surfaces can be found,^40^, ^41^, ^42^ whereas data on the electronic structure is still sparse. The electronic structure of small, neutral copper clusters was investigated in a neon matrix by using spectroscopy techniques within the UV/Vis range.^43^, ^44^, ^45^ In well‐defined gas‐phase experiments, the electronic structure of a ligated CuO^+^ core thought to be responsible for many reactions in nature within copper‐containing enzymes has been characterized,^46^ along with detailed calculations on the bare CuO^+^ ion.^47^ In the closely related Cu(NO_3_)_3_^−^ anion, ligand‐to‐metal charge‐transfer (LMCT) transitions and transitions within the nitrate ligand were observed in the UV region.^48^ Excitation causes the evaporation of a neutral NO_3_ radical, similar to collision‐induced dissociation (CID) experiments, which indicates efficient internal conversion to the electronic ground state.^48^, ^49^, ^50^

Herein, we present an investigation into the electronic structure of copper formate clusters in the gas phase by using UV/Vis spectroscopy along with quantum chemical calculations. We also explore selected photochemical reaction pathways.

## Experimental Section

Mass selected, isotopically enriched copper‐63 formate cluster anions were produced through electrospray ionization (see ref. 31 for details) and investigated at room temperature in the gas phase with a Bruker APEX Qe 9.4 Tesla Fourier‐transformation ion cyclotron resonance (FT‐ICR) mass spectrometer, which is described in more detail elsewhere.^31^, ^51^, ^52^ The trapped clusters were irradiated with an EKSPLA NT342B optical parametric oscillator, which provided pulsed laser light from *λ*=225 to 2600 nm. The obtained photodissociation spectra were corrected for fragmentation caused by blackbody infrared radiative dissociation (BIRD)^53^, ^54^, ^55^, ^56^, ^57^, ^58^ and collisions with the background gas. Photodissociation cross sections were calculated as described in detail before.^59^ The laser power was measured after each mass spectrum was recorded and corrected for transmission through one additional CaF_2_ prism and window. The beam profile was estimated as a homogenous Gaussian beam with a divergence of 0.002 mrad and a beam diameter of 0.005 m at the laser output, along with a constant output angle, leading to uncertainties in the absolute cross‐section calculations. To account for changes in the laser beam profile and alignment upon switching between the different laser stages, a correction factor was applied at the signal to idler beam transition (>710 nm), signal to UV stage (<410 nm), and SF/SH beam transition (<296 nm). Here, the cross section of the signal region was taken as a reference because this was the most reliable value.

For the modeling of the electronic ground‐state properties of copper formate clusters, DFT calculations at the B3LYP/def2TZVP level were used for optimization, based on extensive benchmarking performed previously.^31^ A large number of structures were optimized based on refs. 31 and 33. Herein, we considered several conformers with formate ligands that exhibited different binding motifs (monodentate, bidentate, and bridging bidentate) and orientations on one or more copper centers. The energetically most favorable isomers, with significant structural differences, were then used for further analysis.

For the computational description of the excited states, equation of motion‐coupled cluster singles and doubles (EOM‐CCSD) calculations were performed for small clusters (<150 electrons). The aug‐cc‐pVDZ basis set was used here because it provided results close to the triple‐zeta basis set, see Table S5 in the Supporting Information. All excited states within 6 eV, or at least 12 states (in the case of convergence issues), were considered. For structural optimization in the electronically excited states, the 6‐31+g* basis set was employed. Near conical intersections, the optimization started to oscillate. Therefore, reduced convergence criteria were used (predicted change in energy <2×10^−5^ hartree). To compute electronic transitions in larger clusters, time‐dependent (TD) DFT with the BMK functional was applied, which was chosen based on its performance compared with EOM‐CCSD for copper formate in oxidation state +II, see Table S5 in the Supporting Information. Orbitals participating in electronic transitions were analyzed by using the natural transition orbital (NTO) scheme at the TD‐BMK/aug‐cc‐pVDZ//B3LYP/def2TZVP level of theory. All calculations were carried out with the Gaussian 16 program.^60^ The reported reaction energies were zero‐point corrected.

## Results and Discussion

### Absorption spectra

**Figure 1:**
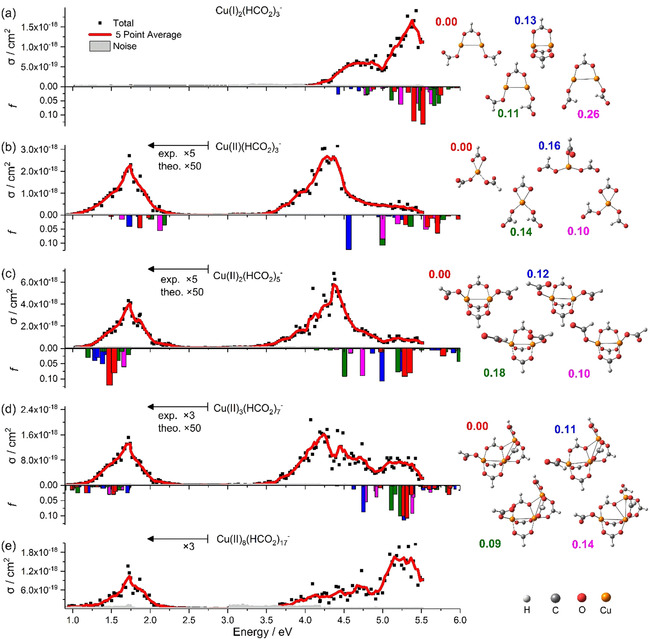
The photodissociation cross section, *σ*, of a) Cu^I^_2_(HCO_2_)_3_^−^, b) Cu^II^(HCO_2_)_3_^−^, c) Cu^II^_2_(HCO_2_)_5_^−^, d) Cu^II^_3_(HCO_2_)_7_^−^, and e) Cu^II^_8_(HCO_2_)_17_^−^, together with the calculated oscillator strength, *f*, of the electronic transitions for selected isomers. Relative energy of isomers is given in eV in the same color used in the bar graph of the oscillator strength within each corresponding subsection. The level of theory is EOM‐CCSD/aug‐cc‐pVDZ//B3LYP/def2TZVP for Cu^I^_2_(HCO)_3_^−^ and Cu^II^(HCO)_3_^−^; TD‐BMK/aug‐cc‐pVDZ//B3LYP/def2TZVP is used for Cu^II^_2_(HCO)_5_^−^ and Cu^II^_3_(HCO)_7_^−^. Experimental cross sections below 2.75 eV are multiplied by a factor of 5 or 3, and theoretical *f* is multiplied by 50 for better legibility.

In Figure 1, spectra of Cu^I^_2_(HCO)_3_^−^, Cu^II^(HCO)_3_^−^, Cu^II^_2_(HCO)_5_^−^, Cu^II^_3_(HCO)_7_^−^, and Cu^II^_8_(HCO)_17_^−^ are shown in the 0.9–5.5 eV region, along with the calculated electronic transitions for selected isomers. The Cu^I^ system, Cu^I^_2_(HCO)_3_^−^, exhibits absorptions only in the UV region, starting at about 4.0 eV. The Cu^II^ systems exhibit two absorption bands in the 1.0–2.4 and 3.5–5.5 eV regions. The intensity of the absorption bands is comparable for all Cu^II^ clusters, with the low‐energy absorption being about five times less intense than that of the high‐energy one.

There is no significant shift in energy observed between systems with one or two copper centers. We can therefore conclude that the bonding interaction between two neighboring copper centers in stoichiometric copper(II) formate clusters is marginal. Instead, the oxidation state of the copper center has the largest influence on the spectra. In the UV spectrum of larger clusters, the electronic transitions appear with a less pronounced maximum and occur deeper within the UV region for the largest case, Cu^II^_8_(HCO)_17_^−^.

The calculated EOM‐CCSD electronic transition energies are shown in Figure 1 a and b. For larger clusters (Figure 1 c and d), TD‐BMK calculations were employed. The calculations reveal that the orientation and binding motif of the formate ligands heavily influence the position and oscillator strength of the bands, as evidenced by the calculated electronic transitions of selected structures. For Cu^I^ compounds and low‐lying states in Cu^II^ species, a very good agreement with the experiments is reached. The higher‐lying states in Cu^II^ compounds, on the other hand, are shifted systematically to higher energies, see, for example, the Cu^II^(HCO)_3_^−^ spectrum in Figure 1 b. Here, multireference effects potentially play a role. Moreover, dynamic effects can further affect the absorption spectra. Considerable energy and intensity shifts, as well as substantial peak broadening, can be expected due to, for example, internal rotation of the formate group or partial opening of a bidentate ligand to a monodentate structure. Zero‐point energy effects,^61^ estimated as 0.2–0.3 eV, might also play a role. Overall, the agreement between experiment and theory is well within the expected range for transition‐metal compounds.

In contrast to the strong effect of the formate ligands on the transitions, the change in the number of copper centers from one to two does not significantly change the position of the transitions, in agreement with experiment. This is also consistent with the previously described extremely weak antiferromagnetic coupling of the unpaired electrons in Cu^II^_2_(HCO)_5_^−^ and the large Cu−Cu distance in copper formate clusters.^31^, ^33^

**Scheme 1:**
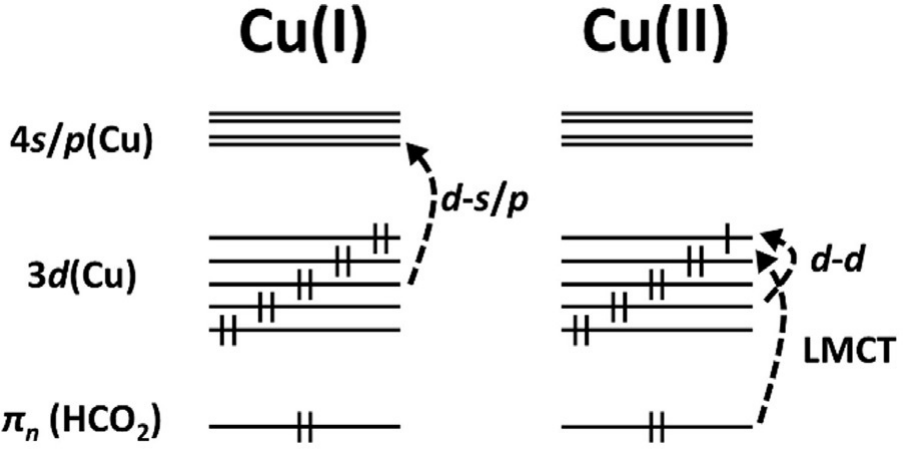
Low‐lying electronic transitions within copper formate complexes for Cu^I^ and Cu^II^.

The observed absorption band structures can be rationalized by the electronic configuration of the copper ion (Scheme 1). In oxidation state +I, a copper ion has a full d shell, whereas there are only nine d electrons for the oxidation state +II. This deeply affects the character of possible excitations.

Copper(I) only affords excitations from the filled d orbital into the empty s/p shells, which corresponds to the band starting at about 4 eV (see Figure 1 a). The hole in the d shell of Cu^II^, however, makes low‐lying d–d transitions and LMCT transitions from the negatively charged formate ligands possible, which correspond to the absorption bands at 1–2 and 3.5–5.5 eV, respectively (see Figure 1 b–e). The d–s/p transitions are substantially blueshifted for Cu^II^ due to the higher number of ligands, which result in more restricted space for the target 4 s*/*p orbitals, and therefore, lie outside the range of our laser system.

**Figure 2:**
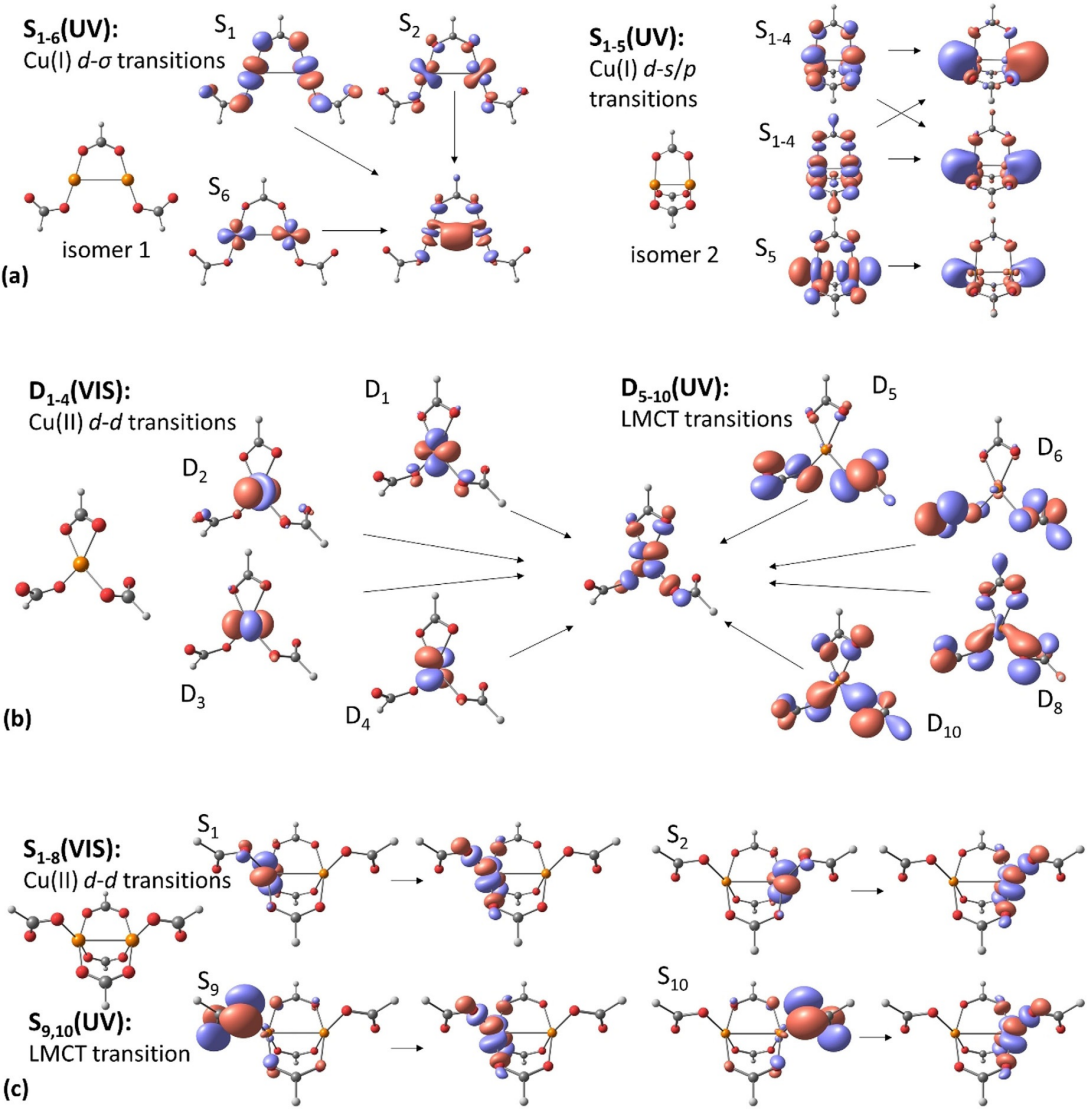
NTOs for selected electronic transitions in a) Cu^I^_2_(HCO_2_)_3_^−^, b) Cu^II^(HCO_2_)_3_^−^, and c) Cu^II^_2_(HCO_2_)_5_^−^ calculated at the TD‐BMK/aug‐cc‐pVDZ//B3LYP/def2TZVP level of theory. Cu−Cu bonds are shown only to guide the eye; for isomer 1 of Cu^I^ and Cu^II^, the Cu–Cu interaction is weak. The shown orbitals are NTOs, that is, they correspond to the initial and final electron orbitals within given excitations. For Cu^I^_2_(HCO_2_)_3_^−^, two isomers are shown. For nearly degenerate transitions, orbitals of different symmetry mix, so only the most important component is shown.

Our analysis of orbitals participating in electronic transitions for Cu^I^_2_(HCO_2_)_3_^−^, Cu^II^(HCO_2_)_3_^−^, and Cu^II^_2_(HCO_2_)_5_^−^ confirms these qualitative arguments (Figure 2). For Cu^I^_2_(HCO_2_)_3_^−^, a transition from copper d‐type orbitals into the unoccupied copper s/p orbitals is observed. In the ground state of Cu^I^_2_(HCO_2_)_3_^−^, the 4 s/p orbitals of the two copper ions are unoccupied. The fully filled d orbitals have a nonbonding character for isomer 1, whereas isomer 2 exhibits a Cu−Cu σ bond. This means a very limited bonding interaction between the two copper centers in S_0_ for isomer 1, whereas isomer 2 has a bonding interaction, which is reflected in the bond length of *d*(Cu−Cu)=3.3 and 2.5 Å in isomers 1 and 2, respectively. Comparing the isomers, one can see why excitation energies within Cu^I^_2_(HCO_2_)_3_^−^ are so sensitive to the orientation of the formate ligands. In isomer 1, two s orbitals form a bonding σ(Cu−Cu) orbital that is compressed by the presence of formate ligands, which extends into the space along the Cu−Cu axis. The single electron is thus confined to a relatively narrow space, which results in a less favorable electronic state. In isomer 2, the s/p orbitals extend freely along the Cu−Cu axis, shifting the first excitation energy by about −1.0 eV. The target orbitals are almost degenerate, irrespective of their phase, showing the independence of both copper ions; see also below for a discussion on the resulting photodynamics.

For Cu^II^(HCO_2_)_3_^−^ (Figure 2 b), four d–d transitions compose the first absorption band, exhibiting a low oscillator strength because they are symmetry forbidden in Cu^II^. In the UV region, the singly occupied d orbital can accept an electron from the nonbonding π orbital of the negatively charged formate ligands. This allows for comparatively intense LMCT transitions. Depending on the orientation of the ligands, the electronic orbitals are more or less confined by the formate ligand, leading again to different excitation energies and oscillator strengths. This also intuitively explains why the open isomer of Cu^II^(HCO_2_)_3_^−^, with only monodentate formate ligands (see Figure 1) has energetically lower‐lying transitions because more space is available for the target orbital. Additionally, the overlap of the ligand orbitals with the copper center is expected to change upon ligand rotation. The observed character of the transitions in Cu^II^(HCO_2_)_3_^−^ is in good agreement with the previously studied Cu^II^(NO_3_)_3_^−^ anion, for which TDDFT calculations predict d–d and LMCT transitions, along with energetically higher transitions on the nitrate ligand.^48^

The singly occupied d orbitals of the copper centers in Cu^II^_2_(HCO_2_)_5_^−^ are independent. This is reflected in the first two participating orbitals of each absorption band illustrated in Figure 2 c, which are energetically nearly degenerate and differ only by the position of the orbital on the right‐ or left‐hand sides of the cluster. Furthermore, the structure has a relatively long bond length of *d*(Cu−Cu)=3.0 Å.

### Photochemistry

**Figure 3:**
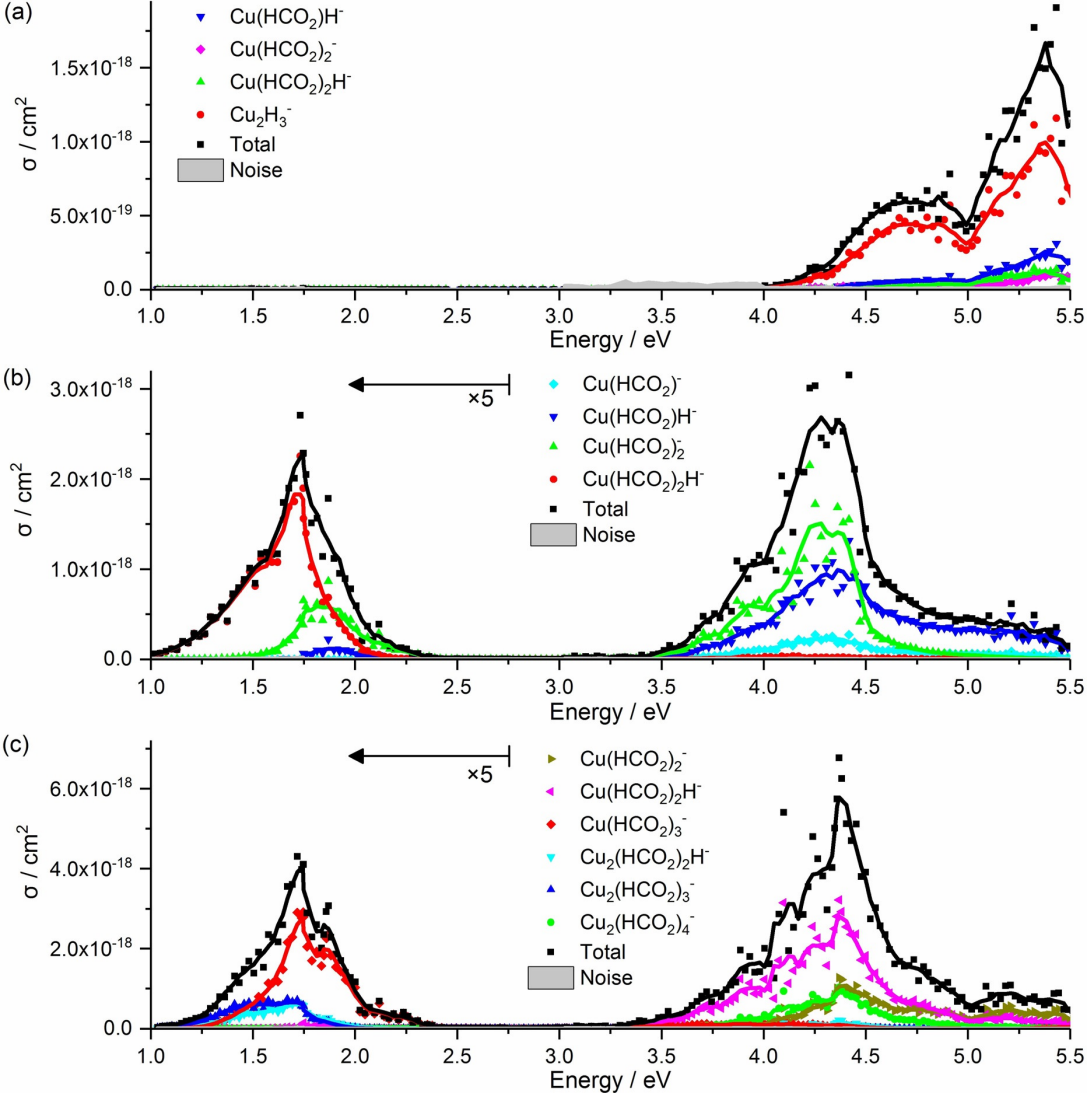
The total dissociation cross section upon laser irradiation of a) Cu^I^_2_(HCO_2_)_3_^−^, b) Cu^II^(HCO_2_)_3_^−^, and c) Cu^II^_2_(HCO_2_)_5_^−^, along with selected partial cross sections of the most important dissociation pathways. Lines represent the respective running average over five data points. The values shown in b) and c) are enlarged by a factor of 5 below 2.75 eV for convenience.

In Figure 3, we analyze the photochemical fragments of copper formate clusters for Cu^I^_2_(HCO_2_)_3_^−^, Cu^II^(HCO_2_)_3_^−^, and Cu^II^_2_(HCO_2_)_5_^−^, as representative examples. The predicted reaction channels for the most intense fragments of the three species are listed in Table 1, together with their experimental appearance energies and calculated reaction energies. In the spectrum of Cu^I^_2_(HCO_2_)_3_^−^, we observe Cu^I^_2_H_3_^−^, Cu^II^(HCO_2_)_2_H^−^, Cu^I^(HCO_2_)_2_^−^, and Cu^I^(HCO_2_)H^−^, reactions (1 a)–(1 d), with roughly constant branching ratio across the absorption band. Reaction (1 a) includes three decarboxylation steps, requiring 2.29 eV, which is the same decomposition pathway as that observed in infrared multiple photon dissociation (IRMPD) experiments.^33^ With UV photons, the clusters can also disproportionate by losing a neutral copper atom and decarboxylate to Cu^II^(HCO_2_)_2_H^−^, reaction (1 b). Alternatively, they could lose a neutral copper formate unit and partially decarboxylate to form Cu^I^(HCO_2_)_2_^−^ or Cu^I^(HCO_2_)H^−^, reactions (1 c) or (1 d), respectively. Reactions (1 b)–(1 d) have not been observed upon IR heating,^33^ which suggests that they require an electronically excited state. The calculated reaction energies are consistently lower than the experimental appearance energies, which confirms that dissociation is energetically feasible with a single photon.

**Table 1:** Appearance energy (*E*_A_ in eV) of the decomposition channels shown in Figure 3 if the signal running average surpasses the noise level, along with the calculated theoretical reaction energy (*E*_theo_), including the highest transition state along the respective reaction pathway (in brackets). Calculated at the B3LYP/def2TZVP level of theory.

Reaction	Reactant	Products	*E*_A_ [eV]	*E*_theo_ [eV]
(1 a)	Cu_2_(HCO_2_)_3_^−^	Cu_2_H_3_^−^+3 CO_2_	4.1	2.29^33^
(1 b)	Cu_2_(HCO_2_)_3_^−^	Cu(HCO_2_)_2_H^−^+Cu+CO_2_	4.4	3.83
(1 c)	Cu_2_(HCO_2_)_3_^−^	Cu(HCO_2_)_2_^−^+Cu(HCO_2_)	4.4	2.04
(1 d)	Cu_2_(HCO_2_)_3_^−^	Cu(HCO_2_)H^−^+Cu(HCO_2_)+CO_2_	4.2	2.97
(2 a)	Cu(HCO_2_)_3_^−^	Cu(HCO_2_)_2_H^−^+CO_2_	1.0	0.66 (1.09)^31^
(2 b)	Cu(HCO_2_)_3_^−^	Cu(HCO_2_)_2_^−^+CO_2+_H	1.3	1.61^31^
(2 c)	Cu(HCO_2_)_3_^−^	Cu(HCO_2_)H^−^+2 CO_2_+H	1.8	2.54^31^
(2 d)	Cu(HCO_2_)_3_^−^	Cu(HCO_2_)^−^+CO_2_+HCOOH	3.5	1.95^31^
(2 d′)	Cu(HCO_2_)_3_^−^	Cu(HCO_2_)^−^+2 CO_2_+H_2_	3.5	1.60^31^
(2 e)	Cu(HCO_2_)_3_^−^	Cu(HCO_2_)_2_^−^+HCO_2_	1.3	1.91
(3 a)	Cu_2_(HCO_2_)_5_^−^	Cu_2_(HCO_2_)_3_^−^+CO_2_+HCOOH	1.0	−0.62 (0.89)^31^
(3 b)	Cu_2_(HCO_2_)_5_^−^	Cu_2_(HCO_2_)_2_H^−^+2 CO_2_+HCOOH	1.0	−0.29 (0.89)^31^
(3 c)	Cu_2_(HCO_2_)_5_^−^	Cu(HCO_2_)_3_^−^+Cu(HCO_2_)_2_	1.2	1.36^31^
(3 d)	Cu_2_(HCO_2_)_5_^−^	Cu(HCO_2_)_2_H^−^+Cu(HCO_2_)_2_+CO_2_	1.6	2.02 (2.45)^31^
(3 e)	Cu_2_(HCO_2_)_5_^−^	Cu(HCO_2_)_2_^−^+Cu(HCO_2_)_2_+CO_2_+H	1.7	2.97^31^
(3 f)	Cu_2_(HCO_2_)_5_^−^	Cu_2_(HCO_2_)_4_^−^+HCO_2_	3.5	2.08

For Cu^II^(HCO_2_)_3_^−^ and Cu^II^_2_(HCO_2_)_5_^−^, fragment branching ratios depend considerably on wavelength (Figure 3 b and c). In the spectrum of Cu^II^(HCO_2_)_3_^−^, all observed fragments, namely, Cu^II^(HCO_2_)_2_H^−^, Cu^I^(HCO_2_)_2_^−^, Cu^I^(HCO_2_)H^−^, and Cu^0^(HCO_2_)^−^, can be explained by previously observed reactions (2 a)–(2 d′) upon IR heating.^31^, ^33^ This points towards a fast internal conversion of the photon energy, followed by decomposition in the ground state. The changing branching ratio could be explained by the amount of available energy, allowing more sequential fragmentation steps for higher photon energies.

For the low‐energy band of Cu^II^_2_(HCO_2_)_5_^−^, mainly the formation of Cu^I^_2_(HCO_2_)_3_^−^, Cu^I^_2_(HCO_2_)_2_H^−^, and Cu^II^(HCO_2_)_3_^−^ is observed, with appearance energies in the range of 1.0–1.2 eV, see reactions (3 a)–(3 c) in Table 1. Additionally, Cu^II^(HCO_2_)_2_H^−^ and Cu^I^(HCO_2_)_2_^−^ are formed through reactions (3 d) and (3 e), albeit with low intensity. For energetic reasons, two photons are required for reactions (3 d) and (3 e), indicating that these ions are secondary products of Cu^II^(HCO_2_)_3_^−^. Reactions (3 a)–(3 e) have all been observed previously in IRMPD experiments,^31^, ^33^ which suggests that internal conversion precedes dissociation. Interestingly, the absorption band starts with the lowest barrier fragment, the formation of formic acid with elimination of CO_2_ (reaction (3 a)). Subsequent CO_2_ elimination from the product is energetically feasible, and becomes more important with increasing photon energy (reaction (3 b)). The entropically favored product, Cu^II^(HCO_2_)_3_^−^, reaction (3 c), requires slightly higher photon energies, in line with experiments, and becomes dominant at the high‐energy end of the absorption band.

In the UV absorption band in the 3.3–5.5 eV region, Cu^II^(HCO_2_)_2_H^−^ is the dominant fragment, followed by Cu^I^(HCO_2_)_2_^−^ (reactions (3 d) and (3 e)). Both reactions require more than 2.4 eV.^31^ Products appearing at the beginning of the fragmentation cascade of Cu^II^_2_(HCO_2_)_5_^−^ are only observed in trace amounts, such as Cu^II^(HCO_2_)_3_^−^ and Cu^I^_2_(HCO_2_)_3_^−^. Only one fragment is observed that is not part of the IRMPD cascade, namely, Cu^I/II^_2_(HCO_2_)_4_^−^, reaction (3 f), which is comparable in intensity to Cu^I^(HCO_2_)_2_^−^. Formation of the formyloxyl radical requires an energy of 2.08 eV. Notably, HCO_2_ might further decompose into H and CO_2_, with a reaction energy of −0.30 eV and a barrier of 0.16 eV. Alternatively, a HOCO unit might be formed with a reaction energy of −0.36 eV. Given that the UV absorption band is due to a LMCT transition, elimination of the neutral formyloxyl radical seems entirely plausible because the alternatives require more extensive rearrangements. The HCO_2_ loss channel after LMCT would also be feasible for Cu^II^(HCO_2_)_3_^−^ through reaction (2 e).

### Photodynamics

**Figure 4:**
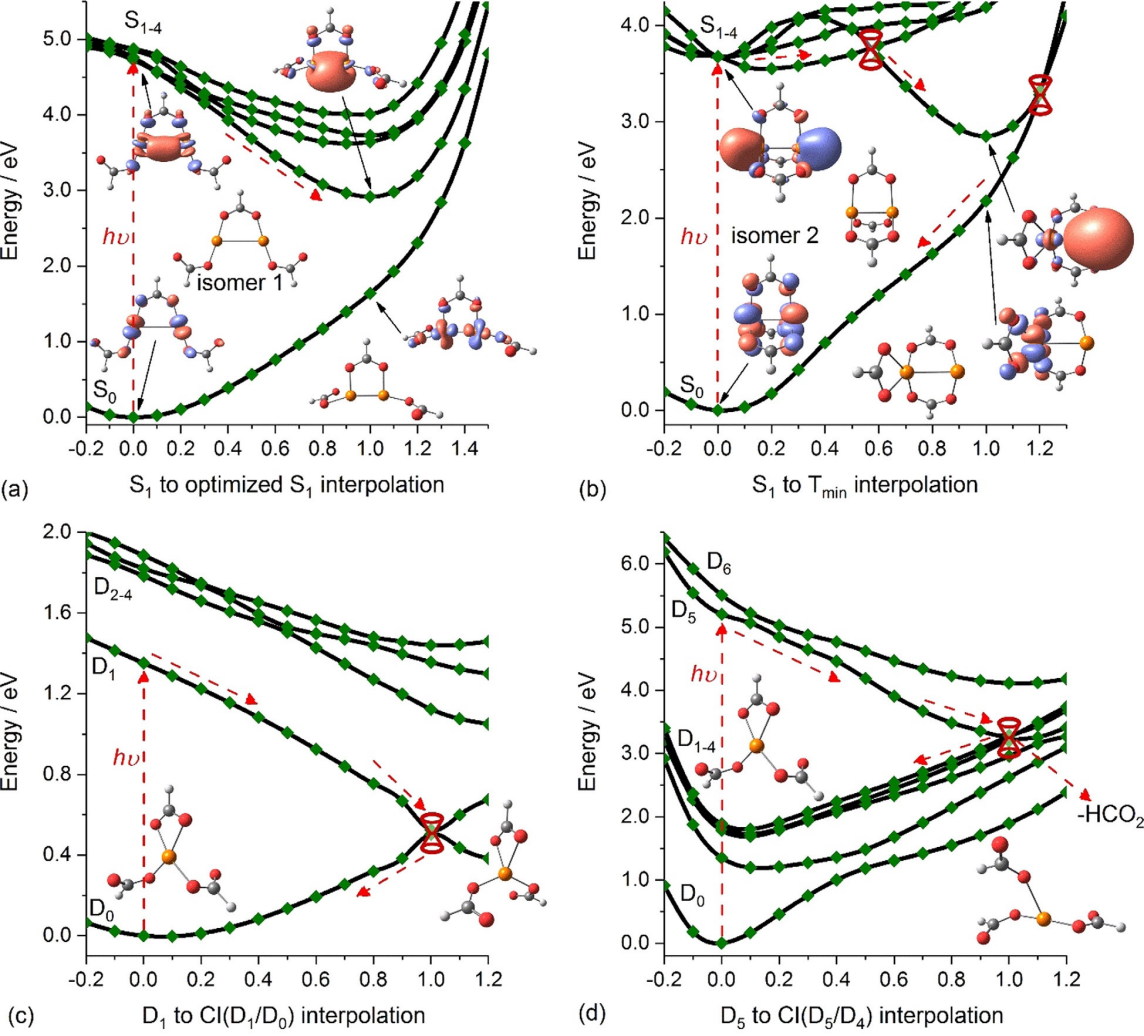
Excited‐state potential‐energy surfaces, along with simplified photochemical reaction coordinates. Interpolation between the Franck–Condon (FC) point towards a) a local minimum within the first excited state of Cu^I^_2_(HCO_2_)_3_^−^ for isomer 1 and b) a local minimum of the lowest lying triplet state in isomer 2. Additionally, NTOs from the ground to the first excited state, along with the corresponding structures in the FC point and minimum, are shown. Interpolation towards c) D_0_/D_1_ and d) D_4_/D_5_ conical intersections in Cu^II^(HCO_2_)_3_^−^, along with the associated structures. The excited states are calculated at the EOM‐CCSD/6‐31+g* level with the FC point optimized at the B3LYP/def2TZVP level of theory. The triplet minimum in isomer 2 of Cu^I^_2_(HCO_2_)_3_^−^ is optimized by using CCSD/6‐31+g*. The NTOs were calculated at the TD‐BMK/aug‐cc‐pVDZ level of theory.

To gain some insight into the photodynamics of Cu^I^_2_(HCO_2_)_3_^−^, we calculated excited‐state potential‐energy curves along the vector connecting the minima of ground and first excited states by using internal coordinates. Figure 4 a,b and Figure S6 in the Supporting Information show the results for isomers 1 and 2, respectively. After excitation, both isomers relax to a local minimum on the excited‐state potential‐energy surface. In isomer 1, a 3 d electron is promoted to a σ(Cu−Cu) orbital, which is half‐filled in S_1_. During geometry optimization, the two monodentate formate ligands rotate out‐of‐plane and the Cu−Cu distance decreases from 3.3 to 2.3 Å, which indicates a strong Cu−Cu interaction due to the occupation of a bonding orbital in S_1_. The resulting Cu−Cu distance is close to the bond length of 2.35 Å in the Cu_2_^−^ ion.^62^ The S_1_ minimum lies 1.8 eV below the S_1_ energy in the FC point. Relaxation through fluorescence into the ground state would be substantially redshifted with respect to the excitation, since the S_1_ minimum lies only 1.3 eV above the respective ground‐state structure. Consequently, a total of (*hv*−1.3 eV) would be available after fluorescence as vibrational energy to support the fragmentation cascade leading to Cu_2_H_3_^−^, which requires 2.29 eV.^33^ Alternatively, the system could reach a conical intersection beyond the S_1_ local minimum at which the extrapolated ground‐ and excited‐state potential‐energy curves nearly coincide, and the entire photon energy would be available for fragmentation on the ground‐state potential‐energy surface. The photodynamics thus rationalize the formation of Cu^I^_2_H_3_^−^, as previously observed in the IRMPD cascade of Cu^I^_2_(HCO_2_)_3_^−^.^33^ No direct mechanism, however, is conceivable for the elimination of a copper atom or copper formate from the excited state for reactions (1 b)–(1 d) from isomer 1.

In isomer 2, the target NTO has an antibonding character, with a possible nonbonding contribution at the FC point. In the S_1_ state, the system relaxes to a local minimum with a slightly elongated Cu−Cu bond (Figure S6 in the Supporting Information), which does not seem to be relevant for the observed photochemistry. Within reach, however, there is a conical intersection seam that is, at most, 0.1 eV above the excitation energy of S_1_ in the FC point. This is easily surpassed with the thermal energy content of the cluster ion before excitation, in addition to surplus energy from excitation into higher states in the experiment. Passing through this conical intersection, the system reaches a minimum with pronounced biradical character (see Figure 4 b), with an electron transferred between the two copper centers. The biradical target structure in Figure 4 b was obtained as a minimum in triplet spin multiplicity (CCSD/6‐31+g*) because geometry optimization of the relevant singlet state at the EOM‐CCSD/6‐31+g* level was less feasible. The excited state after the conical intersection can thus be described as Cu^II^(HCO_2_)_3_Cu^0−^, with the excited state manifold separated by only about 0.6 eV from the ground‐state surface (EOM‐CCSD/6‐31+g*). To reach the biradical minimum, one bridging formate ligand is transferred towards the Cu^II^ center, along with an elongation of the Cu−Cu bond length to 2.7 Å. Furthermore, the Cu−O bonds on the bridging formate ligands are elongated on the Cu^0^ center, providing space for its singly occupied s orbital. In the ground electronic state, the Cu^I^ oxidation state for both copper atoms is maintained. It is conceivable that a neutral Cu^0^ atom can be released from this structure at a cost of 3.18 eV, with respect to the FC point. With the remaining energy, the Cu^II^(HCO_2_)_3_^−^ structure can then sequentially decarboxylate for reaction (1 b), or even afterwards lose a hydrogen radical and another CO_2_ (see reactions (2 b) and (2 c)) to give rise to the Cu^II^(HCO_2_)_2_H^−^, Cu^I^(HCO_2_)_2_^−^, and Cu^I^(HCO_2_)H^−^ fragments. An internal conversion to the ground state becomes accessible through a conical intersection close to this local minimum (Figure 4 b), allowing statistical dissociation on the ground‐state potential‐energy surface in reaction (1a).

Within the photochemistry of Cu^II^(HCO_2_)_3_^−^, all products already appear in the established IRMPD cascade, sharing a sequential reaction pathway (see reactions (2 a)–(2 c)) or branching from it in smaller amounts (see reactions (2 d) and (2 d′)).^31^ This means that they are most likely formed in the electronic ground state. It implies that conical intersections are available to connect the electronically excited states to the ground state. In Figure 4 c and d, we follow relevant excited states along the vector connecting the FC point with D_0_/D_1_ and D_4_/D_5_ conical intersections, again using the internal coordinates. Excitation into the first excited state, D_1_ (Figure 4 c), leads the system barrierlessly into a D_0_/D_1_ conical intersection, with the bidentate ligand rotating around its C−H axis. The trajectory is monotonically downhill, and the full excitation energy is converted into vibrational degrees of freedom, which explains the observed fragmentation channels for the d–d transitions.

For the excitation of Cu^II^(HCO_2_)_3_^−^ into the lowest lying state of the second absorption band, D_5_, the bidentate formate ligand starts dissociating on the way towards a D_4_/D_5_ conical intersection, which is reached again without any barrier (Figure 4 d). Here, the dissociating formate ligand has a relatively long Cu−O bond length of 2.5 Å, compared with the typical bond lengths of 1.9 and 2.1 Å for mono‐ and bidentate formate ligands, respectively, in ground‐state Cu^II^(HCO_2_)_3_^−^. This behavior can be explained by the charge‐transfer character of the LMCT excitation (Figure 2). After an electron is transferred from HCO_2_^−^ to Cu^II^, the now‐neutral HCO_2_ unit is only weakly bound to the cluster. In the vicinity of the conical intersection, the structure of the remaining Cu^I^(HCO_2_)_2_^−^ unit resembles the most stable isomer of the product ion, Cu^I^(HCO_2_)_2_^−^. After passing the D_4_/D_5_ conical intersection, the ion might either follow the dissociation pathway and evaporate a neutral HCO_2_ radical or eventually reach the ground state, as discussed above. HCO_2_ dissociation from Cu^II^(HCO_2_)_3_^−^ in reaction (2 e) is in good agreement with the experimental observation of Cu^I^(HCO_2_)_2_^−^, which exhibits the highest branching ratio in the LMCT band in Figure 3 b. However, HCO_2_ loss for Cu^II^(HCO_2_)_3_^−^ cannot be distinguished from sequential dissociation of CO_2_ and H in reaction (2 b), as observed in the IR experiment.^33^

On the contrary, the formation of Cu_2_(HCO_2_)_4_^−^ from Cu^II^_2_(HCO_2_)_5_^−^ in Figure 3 c is direct evidence that this reaction takes place on the excited‐state potential‐energy surface, since the fragment is not observed in IRMPD and Sustained off‐resonance irradiation (SORI) CID experiments of Cu^II^_2_(HCO_2_)_5_^−^.^31^ All other fragments were also observed in our recent IRMPD experiments.^31^, ^33^ This suggests, once more, that larger Cu^II^ formate clusters can also reach the electronic ground state through conical intersections and redistribute the full photon energy in the system, similar to Cu^II^(HCO_2_)_3_^−^.

Due to this rapid internal conversion, the competitive formation of Cu^II^(HCO_2_)_3_^−^ and Cu^I^_2_(HCO_2_)_3_^−^ from Cu^II^_2_(HCO_2_)_5_^−^, within the d–d band in Figure 3 c, provides further insight into its ground‐state reactivity. Decarboxylation of Cu^II^_2_(HCO_2_)_5_^−^, followed by the formation of formic acid, features a tight transition state. The competitive evaporation of neutral copper diformate Cu^II^(HCO_2_)_2_ can be expected to be entropically favored. However, formic acid formation through reactions (3 a) and (3 b) requires 0.89 eV, compared with 1.36 eV for reaction (3 c), as calculated at the B3LYP/def2TZVP level.^31^ This explains why the channel producing Cu^I^_2_(HCO_2_)_3_^−^, along with its sequential decarboxylation product, Cu^I^_2_(HCO_2_)_2_H^−^, is observed with high selectivity at the start of the absorption band at 1.0 eV, at which the entropically favored evaporation is not yet accessible. Once enough energy is introduced into the ion, the evaporation of neutral copper formate becomes the predominant channel at about 1.5 eV. The production of formic acid can be expected to be highly temperature dependent in catalytic processes, due to the high energy dependence between the two competing reaction channels.

The decomposition trends stay roughly the same for larger Cu^II^ formate clusters (see Figures S4 and S5 and Table S4 in the Supporting Information) and depend mainly on the absorption band. For the d–d transitions in Cu^II^_*n*_(HCO_2_)_2*n*+1_^−^, *n*>2, predominantly the evaporation of neutral copper formate is observed, whereas, for *n=*1 or 2, decarboxylation happens as the first step, as also observed in IRMPD experiments.^31^, ^33^ In the UV band, the dissociation of a formyloxyl radical occurs for Cu^II^_3_(HCO_2_)_7_^−^. However, for a large copper formate cluster, Cu^II^_8_(HCO_2_)_17_^−^, no formyloxyl radical dissociation is observed. Instead, only evaporation of neutral copper formate clusters, similarly to IRMPD,^33^ is recorded, which is consistent with rapid internal conversion followed by statistical decomposition in the electronic ground state.

## Conclusion

The electronic structure of copper formate clusters has been investigated in the gas phase by using UV/Vis spectroscopy between 0.9 and 5.5 eV. For copper centers in oxidation state +I, d–s/p transitions on the copper centers have been observed within the UV region. Copper formate with copper centers in oxidation state +II exhibit copper d–d transitions in the visible/near‐infrared range, along with intense LMCT transitions, which involve the formate ligands and the singly occupied d orbital on the copper center in the UV region.

The number of copper atoms in Cu^II^_*n*_(HCO_2_)_2 *n*+1_^−^ does not change the observed electronic transitions, and thus, provides evidence for the absence of copper–copper bonding, with a singly occupied d orbital at each copper center. The orientation of the formate ligands, however, plays a key role in the spectrum, heavily shifting the intensity and energetic position of the transition.

The photochemistry of Cu^I^_2_(HCO_2_)_3_^−^ depends heavily on the ground‐state structure. Isomer 1, with one bridging formate ligand, most likely dissociates statistically in the electronic ground state, either after fluorescence from a local minimum in S_1_ or by reaching a conical intersection in the vicinity of the S_1_ local minimum. For the paddlewheel structure of isomer 2, Cu−Cu charge transfer initiates the photochemical loss of a neutral copper atom, which competes with statistical dissociation in the electronic ground state. Copper formate clusters in oxidation state +II are able to relax ultrafast back to the ground state through conical intersections in both excitation bands, and the redistributed energy is available for statistical dissociation. However, in the UV region, LMCT makes photochemical formation of formyloxyl radicals possible. For large copper formate clusters, no formyloxyl radical loss is observed, which indicates that the photon energy is redistributed into vibrational degrees of freedom. Through the efficient energy redistribution by conical intersections, the selectivity in the formation of formic acid can be tuned via the photon energy in the d–d transition of Cu^II^_2_(HCO_2_)_5_^−^. Higher energies favor entropically preferred dissociation pathways with loose transition states, such as the evaporation of Cu^II^(HCO_2_)_2_. A large temperature dependence in the formic acid yield is therefore expected under catalytic conditions.

## Conflict of interest

The authors declare no conflict of interest.

## Supporting information

As a service to our authors and readers, this journal provides supporting information supplied by the authors. Such materials are peer reviewed and may be re‐organized for online delivery, but are not copy‐edited or typeset. Technical support issues arising from supporting information (other than missing files) should be addressed to the authors.

Supplementary
