# Supplementary material for: UV/Vis Spectroscopy of Copper Formate Clusters: Insight into Metal‐Ligand Photochemistry
Source: Chemistry. 2020 Jun 18;26(37):8286–95. doi: 10.1002/chem.202000280 (PMC7384192; doi:10.1002/chem.202000280)
Supplement: Supplementary file 1 — Supplementary [file CHEM-26-8286-s001.pdf]

Chemistry–A European Journal

Supporting Information

UV/Vis Spectroscopy of Copper Formate Clusters: Insight into Metal-Ligand Photochemistry

Tobias F. Pascher, Milan Ončák,* Christian van der Linde, and Martin K. Beyer*[a]

Supporting Information

for

UV/VIS spectroscopy of Copper Formate Clusters: Insight into Metal-Ligand Photochemistry

Tobias F. Pascher, Milan Ončák, Christian van der Linde and Martin K. Beyer**

Institut für Ionen und Angewandte Physik, Universität Innsbruck, Technikerstraße 25, 6020 Innsbruck, Austria

Corresponding Author

*E-mail: Milan.Oncak@uibk.ac.at, Martin.Beyer@uibk.ac.at

Experiments and expected reaction pathways

We measured the photo dissociation cross section for $Cu(I)(HCO_2)_2^-$. However, $Cu(I)(HCO_2)_2^-$ does not fragment substantially with rather long irradiation times of up to 5 s and additional fragmentation through laser irradiation is only within the ‰ regime. The dominant fragmentation of $Cu(I)(HCO_2)_2^-$ takes place through BIRD and collisions with the background gas in the % regime, which could not be entangled completely due to heating of the cell with the laser beam. Furthermore, $Cu(I)(HCO_2)H^-$ photo-detaches around 4.2 eV. Due to these problems, the experiment can only suggest that $Cu(I)(HCO_2)_2^-$ photodissociates in the UV region with a dissociation cross section in the regime of 10^{-21} cm^2. It is possible that $Cu(I)(HCO_2)_2^-$ strongly fluoresces as the excited states within Cu(+I) formate exhibit local minima to be reached from the Franck Condon region.

The dissociation cross section of $Cu(I)_2(HCO_2)_3^-$, $Cu(II)(HCO_2)_3^-$, $Cu(II)_2(HCO_2)_5^-$, $Cu(II)_3(HCO_2)_7^-$ and $Cu(II)_8(HCO_2)_{17}^-$ including all partial cross sections are illustrated in Figures S1–5 along with their predicted reaction pathway with the calculated reaction energy in Tables S1–4.

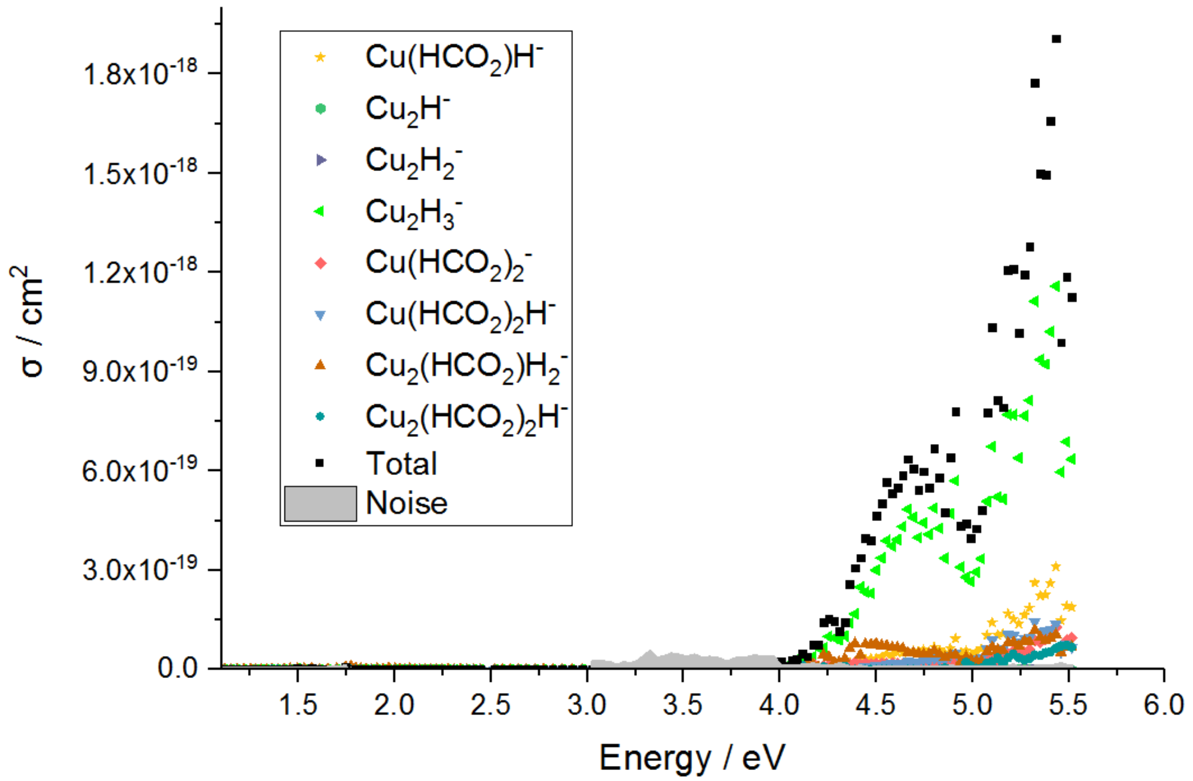

FIGURE S1. Total dissociation cross section upon laser irradiation of Cu(I)$_2$(HCO$_2$)$_3^-$ along with partial cross sections.

TABLE S1. Reaction energies of Cu(I)$_2$(HCO$_2$)$_3^-$ decomposition E_{theo} of the fragments seen in Figure S1. Calculated at the B3LYP/def2TZVP level of theory. The channel leading to $Cu_2H_2^-$ is predicted to be a multi-photon process consistent with the low intensity in the experiment.

Reactant	Product	E_{theo} /eV
$Cu_2(HCO_2)_3^-$	$Cu(HCO_2)H^- + Cu(HCO_2) + CO_2$	2.97
	$Cu_2H^- + H_2 + 3\ CO_2$	3.32
	$Cu_2H_2^- + H + 3\ CO_2$	5.74
	$Cu_2H_3^- + 3\ CO_2$	2.29[1]
	$Cu(HCO_2)_2H^- + Cu + CO_2$	3.83
	$Cu(HCO_2)_2^- + Cu + CO_2 + H$	4.79
	$Cu(HCO_2)H^- + Cu + 2\ CO_2 + H$	5.72
	$Cu_2(HCO_2)H_2^- + 2\ CO_2$	1.27[1]
	$Cu_2(HCO_2)_2H^- + CO_2$	0.32[1,2]

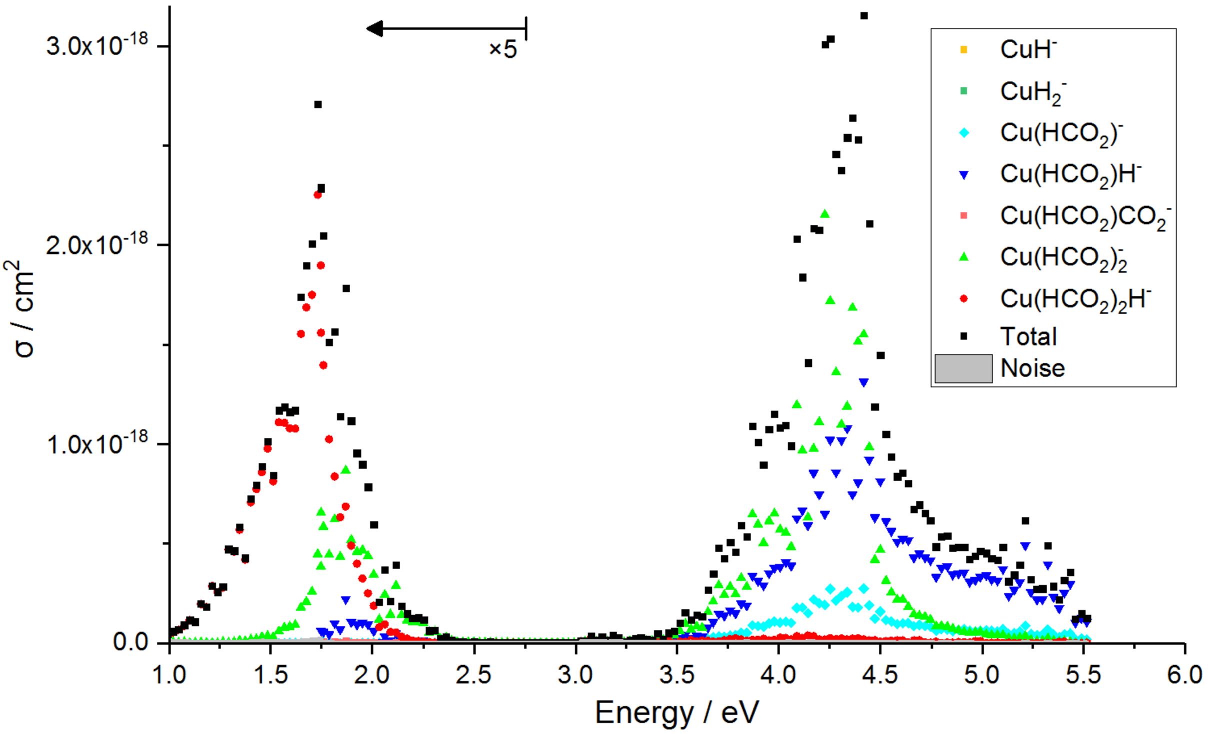

FIGURE S2. Total dissociation cross section upon laser irradiation of Cu(II)$(HCO_2)_3^-$ along with partial cross sections. Below 2.75 eV, the intensity is enlarged by a factor of 5 for visibility.

TABLE S2. Reaction energies of Cu(II)$(HCO_2)_3^-$ decomposition E_{theo} of the fragments seen in Figure S2. Calculated at the B3LYP/def2TZVP level of theory.

Reactant	Product	E_{theo} /eV
$Cu(HCO_2)_3^-$	CuH^- + 3 CO_2 +H_2	2.90
	CuH_2^- + 3 CO_2 +H	3.87
	$Cu(HCO_2)^-$ + 2 CO_2 + H_2	1.60[2]
	$Cu(HCO_2)^-$ + HCOOH+ CO_2	1.95[2]
	$Cu(HCO_2)H^-$ + 2 CO_2 + H	2.54[2]
	$Cu(HCO_2)CO_2^-$ + CO_2 + H_2	0.82[2]
	$Cu(HCO_2)CO_2^-$ + HCOOH	1.17[2]
	$Cu(HCO_2)_2^-$ + CO_2 + H	1.61[2]
	$Cu(HCO_2)_2H^-$ + CO_2	0.66[2]
	$Cu(HCO_2)_2^-$ + HCO_2	1.91

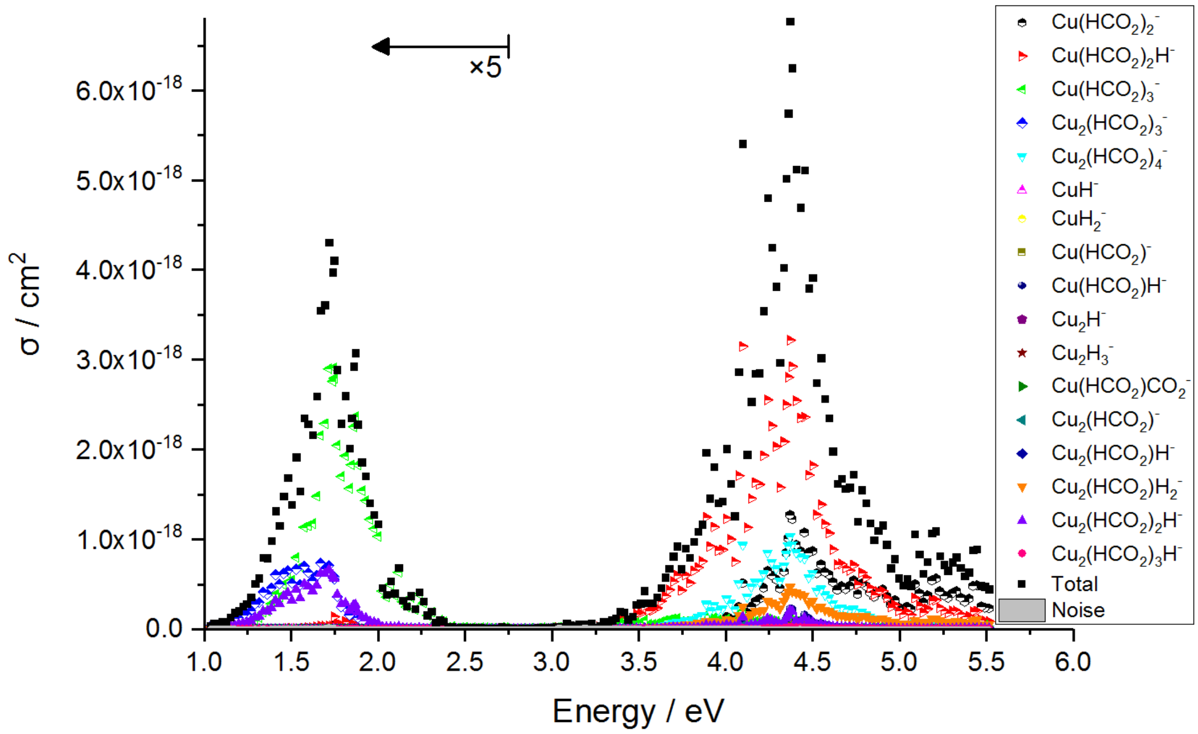

FIGURE S3. Total dissociation cross section upon laser irradiation of $Cu(II)_2(HCO_2)_5^-$ along with partial cross sections. Below 2.75 eV, the intensity is enlarged by a factor of 5 for visibility.

TABLE S3. Reaction energies of $Cu(II)_2(HCO_2)_5^-$ decomposition E_{theo} of the fragments seen in Figure S3. Calculated at the B3LYP/def2TZVP level of theory. The channel leading to $Cu_2(HCO_2)H^-$ and $Cu_2(HCO_2)^-$ are predicted to be a multi-photon process consistent with the low intensity in the experiment. There are many products plausible for these two fragments and two examples are listed. It is unlikely that they access the energetically lowest product channel, as the products are missing in the spectrum of $Cu_2(HCO_2)_3^-$.

	Product	E_{theo} /eV
$Cu_2(HCO_2)_5^-$	$Cu_2(HCO_2)_3^- + CO_2 + HCOOH$	-0.62[2]
	$Cu(HCO_2)_3^- + Cu(HCO_2)_2$	1.36[2]
	$Cu_2(HCO_2)_4^- + HCO_2$	2.08
	$Cu_2(HCO_2)_3H^- + HCO_2 + CO_2$	2.56
	$Cu_2(HCO_2)H^- + HCOOH + HCO_2 + 2\ CO_2$	4.37
	$Cu_2(HCO_2)H^- + H_2 + H + 4\ CO_2$	3.72
	$Cu_2(HCO_2)^- + H + HCOOH + HCO_2 + 2\ CO_2$	6.46
	$Cu_2(HCO_2)^- + 2\ H_2 + 4\ CO_2$	1.31

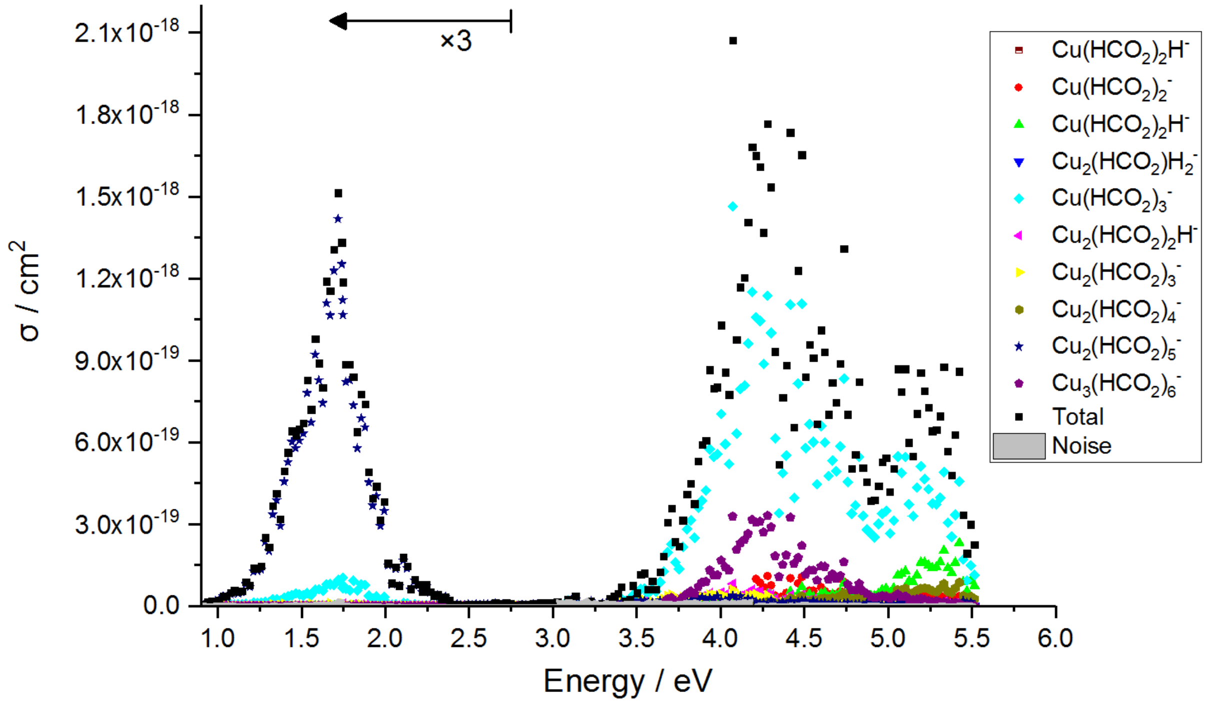

FIGURE S4. Total dissociation cross section upon laser irradiation of Cu(II)$_3$(HCO$_2$)$_7^-$ along with partial cross sections. Below 2.75 eV, the intensity is enlarged by a factor of 3 for visibility.

TABLE S4. Reaction energies of Cu(II)$_3$(HCO$_2$)$_7^-$ decomposition E_{theo} of the fragments seen in Figure S4. Calculated at the B3LYP/def2TZVP level of theory.

Reactant	Product	E_{theo} /eV
$Cu_3(HCO_2)_7^-$	$Cu(HCO_2)_3^- + Cu_2(HCO_2)_4$	1.16[1]
	$Cu_2(HCO_2)_5^- + Cu(HCO_2)_2$	1.10[1]
	$Cu_3(HCO_2)_6^- + HCO_2$	2.05

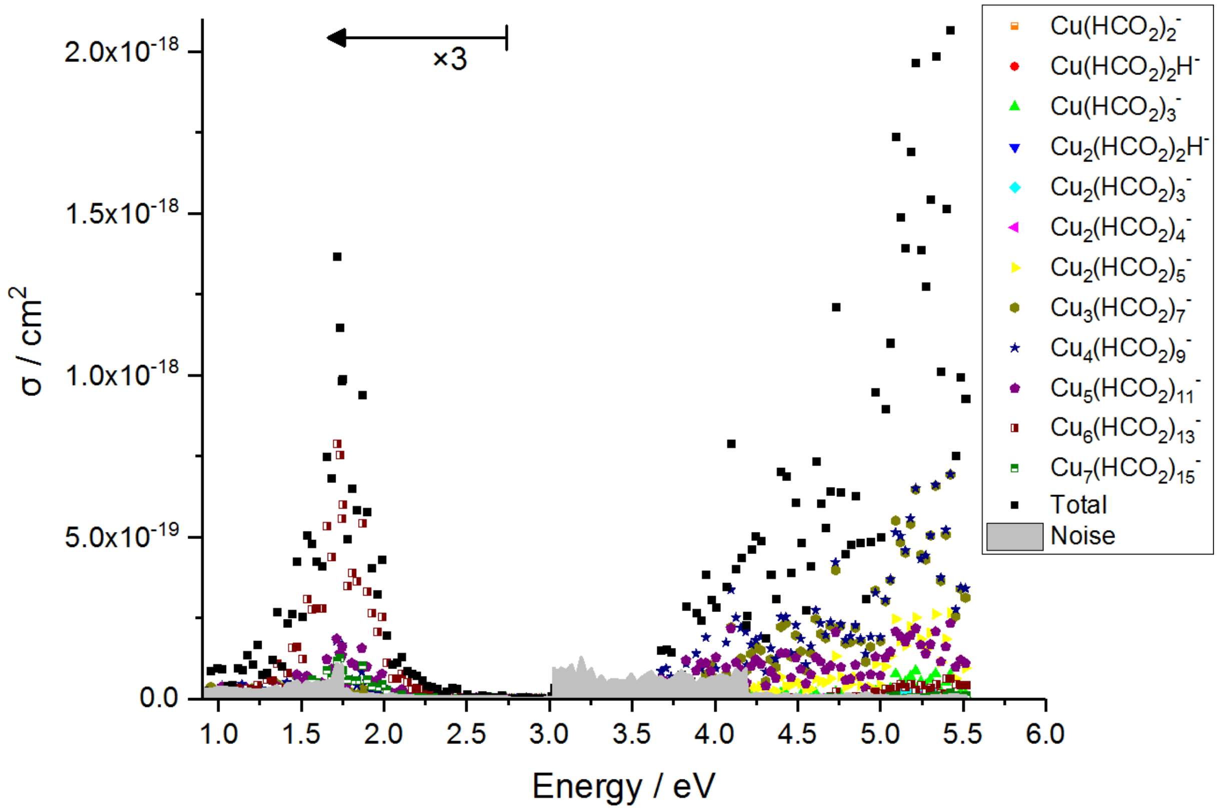

FIGURE S5. Total dissociation cross section upon laser irradiation of $Cu(II)_8(HCO_2)_{17}^-$ along with partial cross sections. Below 2.75 eV, the intensity is enlarged by a factor of 3 for visibility. Primary decomposition takes place through evaporation of neutral copper formate clusters.

Excited State Potential Energy Surface

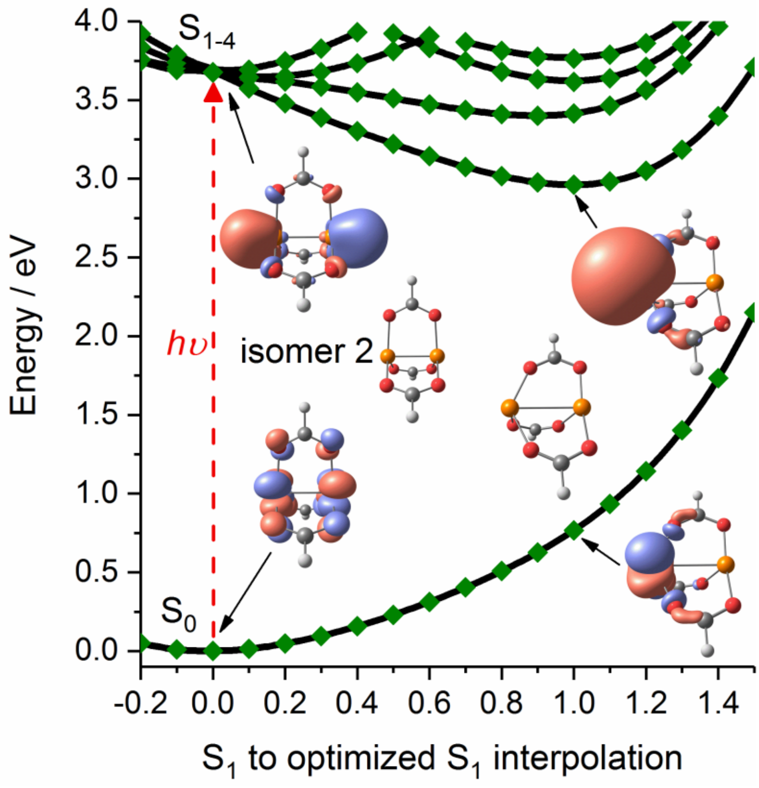

FIGURE S6. Interpolation between the Franck-Condon point towards a local minimum within the first excited state of $Cu(I)_2(HCO_2)_3^-$ for isomer 2 along with the natural transition orbitals of the first excited state and structures in the Franck-Condon point and minimum. The first four excited states of the same spin multiplicity, are calculated at the EOM-CCSD/6-31+g* level with the FC point optimized at the B3LYP/def2TZVP level of theory. The natural transition orbitals were calculated at the TD-BMK/aug-cc-pVDZ level of theory.

Theoretical methods and benchmarking

For energy benchmarking of basis sets and methods for two reactions observed with copper formate, see Ref. [2]. Based on those results, the B3LYP functional with the def2TZVP basis set is chosen for geometry optimization as it agrees well with higher-level methods. Benchmarking of excited states for Cu(II)$(HCO_2)_3^-$ is depicted in Table S5. The aug-cc-pVDZ basis set is chosen because it is very close to the aug-cc-pVTZ results with the average absolute difference $\overline{|\Delta E|} \approx 0.03$ eV. However, results of TD-DFT methods can only reproduce EOM-CCSD values with an average error of $\overline{|\Delta E|} = 0.4$–$1.0$ eV and predict states with significant spin contamination. Therefore, the TD-BMK method is used only for larger clusters when EOM-CCSD becomes computationally unfeasible. It has the lowest discrepancy with respect to the EOM-CCSD method with the average absolute difference of $\overline{|\Delta E|} = 0.38$ eV, see Table S5.

Table S5. Benchmarking of basis sets and *ab initio* methods for excited state calculations within Cu(II)$(HCO_2)_3^-$ using geometry optimized structures on the B3LYP/def2TZVP level of theory showing the energy E /eV along with the corresponding oscillator strength f for the first ten excited states. For comparison, the average absolute discrepancy of the transition energy E_i calculated with the method i (i = 6-31+g*, aug-cc-pVDZ, aug-cc-pVTZ) compared to EOM-CCSD $\overline{|E_{\mathrm{CCSD}} - E_i|}$ along with the average absolute difference of basis set j (j = 6-31+g*, aug-cc-pVTZ) towards the transition energy E_j with the basis set aug-cc-pVDZ $\overline{|E_{\mathrm{DZ}} - E_j|}$ is given.

	EOM-CCSD				BMK						BhandHLYP						CAM-B3LYP					
	6-31+g*		aug-cc-pVDZ		6-31+g*		aug-cc-pVDZ		aug-cc-pVTZ		6-31+g*		aug-cc-pVDZ		aug-cc-pVTZ		6-31+g*		aug-cc-pVDZ		aug-cc-pVTZ	
	E	*f*	*E*	*f*	*E*	*f*	*E*	*f*	*E*	*f*	*E*	*f*	*E*	*f*	*E*	*f*	*E*	*f*	*E*	*f*	*E*	*f*
1B	1.35	0.000	1.50	0.000	1.29	0.000	1.30	0.000	1.31	0.000	1.36	0.000	1.41	0.000	1.43	0.000	1.59	0.002	1.60	0.001	1.63	0.001
2B	1.78	0.001	1.87	0.001	1.42	0.000	1.48	0.000	1.51	0.000	1.42	0.000	1.47	0.000	1.52	0.000	1.63	0.000	1.64	0.000	1.68	0.001
3B	1.82	0.000	1.93	0.000	1.49	0.000	1.51	0.000	1.54	0.000	1.58	0.000	1.63	0.000	1.67	0.000	1.85	0.000	1.85	0.000	1.89	0.000
1A	1.89	0.000	1.98	0.000	1.55	0.000	1.57	0.000	1.60	0.000	1.64	0.000	1.68	0.000	1.72	0.000	1.93	0.001	1.93	0.001	1.96	0.001
2A	5.20	0.032	5.29	0.035	4.70	0.013	4.93	0.013	4.96	0.015	5.67	0.019	5.95	0.020	6.00	0.022	3.68	0.013	3.86	0.014	3.87	0.015
4B	5.51	0.042	5.58	0.042	4.96	0.000	5.15	0.000	5.20	0.001	6.03	0.028	6.21	0.000	6.24	0.001	3.99	0.001	4.15	0.000	4.16	0.001
3A	5.54	0.010	5.62	0.011	4.84	0.001	5.04	0.001	5.08	0.001	6.07	0.002	6.20	0.001	6.23	0.001	3.85	0.001	4.00	0.001	4.02	0.001
4A	5.65	0.076	5.71	0.064	5.26	0.087	5.45	0.074	5.45	0.074	6.12	0.047	6.36	0.048	6.40	0.045	4.27	0.112	4.42	0.105	4.41	0.104
5B	5.66	0.001	5.72	0.001	5.10	0.023	5.30	0.023	5.32	0.027	6.15	0.005	6.28	0.028	6.32	0.030	4.16	0.044	4.30	0.044	4.30	0.047
6B	5.76	0.003	5.79	0.000	5.28	0.032	5.49	0.026	5.52	0.024	6.23	0.016	6.48	0.017	6.45	0.013	4.27	0.009	4.44	0.010	4.45	0.008
$\overline{\vert E_{\mathrm{DZ}} - E_j\vert}$	0.08	-	-	-	0.13	-	-	-	0.03		0.14	-	-	-	0.03	-	0.10	-	-	-	0.02	
$\overline{\vert E_{\mathrm{CCSD}} - E_i\vert}$	-	-	-	-	0.43	-	0.38	-	-	-	0.38	-	0.48	-	-	-	0.95	-	0.90	-	-	-

Literature

[1] T. F. Pascher, M. Ončák, C. van der Linde, M. K. Beyer, *ChemistryOpen* **2019**, *8*, 1453-1459.

[2] T. F. Pascher, M. Ončák, C. van der Linde, M. K. Beyer, *ChemPhysChem* **2019**, *20*, 1420.

Cartesian coordinates of optimized ions and molecules (in Å, calculated at the B3LYP/def2TZVP level unless noted otherwise) along with electronic energies (in Hartree) including zero point energy. The remaining structures can be found in the supporting information of Refs. [1,2].

Cu(HCO2)3- Iso. 1
E=-2208.389679
H 0.000000 -0.000000 4.031746
C 0.000000 -0.000000 2.929280
O 0.144356 -1.087879 2.316270
O -0.144356 1.087879 2.316270
Cu 0.000000 -0.000000 0.523972
O 0.000000 -1.487685 -0.652551
O 0.000000 1.487685 -0.652551
C 0.729627 -1.607774 -1.717445
C -0.729627 1.607774 -1.717445
O 0.776951 -2.594235 -2.430716
O -0.776951 2.594235 -2.430716
H 1.346458 -0.717071 -1.960668
H -1.346458 0.717071 -1.960668

Cu(HCO2)3- Iso. 2
E= -2208.391103
Cu 0.315991 -0.696103 -0.099124
O -0.942967 0.706328 -0.122990
C -0.686958 1.975342 0.002475
H 0.384929 2.212185 0.132969
O -1.528864 2.857598 -0.017004
O 1.060211 -2.616092 -0.164279
O 1.908096 0.329132 0.152665
C 3.093726 -0.190491 0.258717
O 4.123333 0.442487 0.419579
H 3.123635 -1.295949 0.192069
C -0.111785 -3.059068 -0.333213
H -0.252006 -4.147671 -0.434482
O -1.100487-2.296721 -0.386773

Cu(HCO2)3- Iso. 3
E= -2208.394947
Cu -0.000013 0.080893 0.000006
C -0.000227 2.478002 -0.000007
H -0.000326 3.581113 -0.000013
O -1.046195 1.866856 -0.334719
O 1.045850 1.867046 0.334712
O -1.423231 -1.195883 -0.293911
C -2.087562 -1.428811 0.778471
H -2.913568 -2.156033 0.618377
O -1.905610 -0.950606 1.892234
O 1.423433 -1.195626 0.293936
C 2.087805 -1.428446 -0.778443
O 1.905768 -0.950286 -1.892211
H 2.913941 -2.155519 -0.618341

Cu(HCO2)3- Iso. 4
E= -2208.388929
C -2.901269 -0.332892 -0.077037
O -1.797905 -0.901673 0.309550
Cu -0.038221 -0.425843 -0.034740
O 1.703767 -1.055588 -0.043889
C 2.837009 -0.420788 0.027388
O 3.933378 -0.944199 0.057202
O -4.022131 -0.756675 0.125825
H -2.744904 0.613323 -0.632821
H 2.734830 0.681381 0.062029
O -0.101693 1.272384 -0.980149
C 0.125262 2.267416 -0.194631
H 0.081797 3.241081 -0.725382
O 0.368416 2.227159 1.002624

Cu2(HCO2)3- Iso. 1
E= -3849.053932
C 0.000000 0.000000 2.576920
H 0.000000 0.000000 3.679133
Cu 0.000000 1.652720 0.230393
Cu -0.000000 -1.652720 0.230393
O 0.000000 1.132641 2.041387
O -0.000000 -1.132641 2.041387
O 0.000000 2.285382 -1.524936
O -0.000000 -2.285382 -1.524936
C 0.000000 3.556168 -1.726797
C -0.000000 -3.556168 -1.726797
O 0.000000 4.460068 -0.901513
O -0.000000 -4.460068 -0.901513
H 0.000000 3.804482 -2.810446
H -0.000000 -3.804482 -2.810446

Cu2(HCO2)3- Iso. 2
E= -3849.049191
Cu 1.269869 0.000000 0.000000
Cu -1.269869 0.000000 0.000000
O 1.131773 0.000000 -2.046717
O 1.131773 1.772508 1.023358
O 1.131773 -1.772508 1.023358
O -1.131773 0.000000 -2.046717
O -1.131773 -1.772508 1.023358
O -1.131773 1.772508 1.023358
C 0.000000 0.000000 -2.587291
H 0.000000 0.000000 -3.697009
C 0.000000 2.240660 1.293646
H 0.000000 3.201704 1.848505
C 0.000000 -2.240660 1.293646
H 0.000000 -3.201704 1.848505

Cu2(HCO2)3- Iso. 3
E= -3849.050048
C -1.617004 2.639744 0.000000
O -2.403295 3.571909 0.000000
O -0.323666 2.760615 0.000000
Cu 0.986834 1.469353 0.000000
Cu 0.000000 -1.419171 -0.000000
O -1.858903 -1.271738 0.000000
C -2.559965 -2.352230 0.000000
O -2.167090 -3.511222 -0.000000
O 2.599960 0.485831 -0.000000
C 2.750716 -0.759443 -0.000000
O 1.876366 -1.656472 -0.000000
H -1.991990 1.596874 0.000000
H 3.794554 -1.111134 -0.000000
H -3.650205 -2.140830 0.000000

Cu2(HCO2)3- Iso. 4

E= -3849.044326
cu -1.553521 -0.667112 0.190117
cu 1.650544 -0.518310 -0.168866
c 0.152875 -2.949326 -0.021790
o -0.993175 -2.463833 0.123588
o 1.249754 -2.358510 -0.152628
o 2.435118 1.140432 -0.279924
c 2.170476 2.238619 0.357591
o 2.857916 3.243247 0.343198
o -2.456360 0.926166 0.367337
c -2.494021 1.944359 -0.434615
o -3.280444 2.870598 -0.363822
h 0.202519 -4.049602 -0.036699
h -1.724545 1.937206 -1.237133
h 1.229921 2.222922 0.948432

Cu2(HCO2)5- Iso. 1
E=-4227.500022
C -0.169863 0.016429 -2.481485
O -1.285276 0.238053 -1.948706
Cu -1.606056 0.462397 -0.000225
O -3.203326 -0.588253 -0.000016
C -3.166454 -1.876574 -0.000080
O -2.190232 -2.605063 0.000002
Cu 1.442632 0.011898 0.000310
O 1.353903 1.980562 0.001609
C 0.320192 2.683811 0.000548
O -0.875534 2.312445 -0.000757
O 0.961090 -0.081911 -1.956678
O 2.106043 -1.822497 -0.000820
C 3.370845 -1.640892 -0.000721
O 3.928309 -0.540516 0.000007
O 0.960536 -0.084383 1.956960
C -0.170427 0.015474 2.481450
O -1.285353 0.238947 1.948412
H -0.194922 -0.105514 -3.578487
H 0.483997 3.774594 0.000789
H -4.189883 -2.313069 -0.000206
H -0.195998 -0.106787 3.578405
H 3.969055 -2.572362 -0.001343

Cu2(HCO2)5- Iso. 2
E=-4227.497680
H 2.576395 1.618170 -0.197927
O 0.181867 2.612260 0.167382
C -0.987905 2.967504 0.173780
H -1.242599 4.045501 0.242694
O -2.030910 2.218608 0.107607
Cu -1.665140 0.335779 -0.024580
O -1.233018 0.456148 -1.966914
O -1.455061 0.138250 1.943854
O -2.192687 -1.566964 -0.211576
C -1.386224 -2.521562 -0.299162
O -0.136878 -2.486178 -0.258867
C -0.428222 -0.337638 2.489359
O 0.610363 -0.798137 1.966349
C -0.113353 0.165738 -2.454623
O 0.890697 -0.336371 -1.900473
Cu 1.062732 -0.917344 0.010383
O 2.839216 -0.345814 0.227997
C 3.330602 0.840780 0.020849
O 4.518405 1.108630 0.068678
H -0.001205 0.381483 -3.530880
H -0.450961 -0.352518 3.592645
H -1.837145 -3.519658 -0.428368

Cu2(HCO2)5- Iso. 3
E=-4227.500631
C -0.163998 0.892471 2.390939
O -1.236100 0.455380 1.911737
Cu -1.491614 -0.086268 0.000299
O -1.013573 -0.623148 -1.882829
C 0.147078 -0.680465 -2.343396
O 1.232459 -0.338561 -1.809354
Cu 1.425365 0.596819 -0.056398
O 0.583723 2.252858 -0.747972
C -0.635889 2.532192 -0.824544
O -1.618405 1.807286 -0.553845
O 0.947056 1.058944 1.834968
O 2.965120 -0.348679 0.495204
C 3.300683 -1.565795 0.196385
O 4.375062 -2.076853 0.455637
O -1.727547 -1.959337 0.510700
C -3.000190 -2.088141 0.464611
O -3.791971 -1.194619 0.161807
H -0.203379 1.171926 3.457808
H -0.872418 3.549458 -1.177461
H 2.515330 -2.145697 -0.327392
H 0.236407 -1.090235 -3.363881
H -3.367414 -3.099177 0.725427

Cu2(HCO2)5- Iso. 4
E= -4227.504363
Cu 0.137794 -1.496112 -0.000000
Cu 0.035013 1.500805 0.000000
O -0.067127 1.131744 1.970118
C -0.067127 -0.002491 2.502861
O 0.011020 -1.134114 1.970464
H -0.145754 -0.004955 3.604165
O -0.067127 1.131744 -1.970118
C -0.067127 -0.002491 -2.502861
O 0.011020 -1.134114 -1.970464
O 2.065027 -1.058335 -0.000000
C 2.568249 0.088027 0.000000
O 1.988141 1.197354 0.000000
H 3.670364 0.125403 0.000000
H -0.145754 -0.004955 -3.604165
O -1.618907 -2.353232 -0.000000
C -1.329833 -3.599530 -0.000000
O -0.191471 -4.068905 -0.000000
H -2.211667 -4.270125 -0.000000
O -1.774923 2.239341 0.000000
C -1.570345 3.502249 0.000000
O -0.466404 4.047533 0.000000
H -2.495500 4.111822 0.000000

Cu3(HCO2)7- Iso. 1
E= -6246.603900
H 4.065487 3.584274 -0.966743
C 3.706453 2.607725 -0.606873
O 3.859328 1.589319 -1.330218
O 3.146538 2.528959 0.516519
Cu 2.926487 0.479635 0.155068
O 3.520079 -1.252733 -0.461184
C 2.845647 -2.327079 -0.496106
H 3.441935 -3.210449 -0.792669
O 1.648429 -2.504772 -0.249893
O 2.452520 -0.038733 1.983605
C 1.391921 -0.482141 2.487026
O 0.352497 -0.904715 1.936612
H 1.377395 -0.505018 3.590209
O 0.719644 0.624103 -0.470977
C 0.085889 1.659217 -0.827066
H 0.706119 2.518514 -1.121998
O -1.146177 1.820696 -0.885067
Cu -0.074493 -1.227020 0.038288
Cu -2.547983 0.530720 -0.210018
O -0.589421 -1.550063 -1.835308
C -1.637739 -1.153229 -2.395209
O -2.539250 -0.412030 -1.943898
H -1.783053 -1.507493 -3.428940
H -3.372318 -2.920508 1.367934
O -3.366570 -1.046664 0.694199
O -1.567154 -2.423602 0.686829
C -2.760826 -2.122065 0.912603
O -2.871040 1.661354 1.348224
C -3.921068 2.314392 1.015755
H -4.274197 3.028195 1.785186
O -4.524341 2.198238 -0.050763

Cu3(HCO2)7- Iso. 2
E= -6246.599735
Cu 2.903024 0.528013 0.193167
Cu -0.091337 -1.179509 0.237225

Cu -2.518927 0.368183 -0.709725
H 4.210697 3.166962 -1.674859
C 3.798217 2.336235 -1.082449
O 4.037315 1.150260 -1.428920
O 3.091302 2.586086 -0.072106
O 2.184657 0.584121 2.017822
C 1.055331 0.321137 2.494031
O 0.076052 -0.258556 1.972215
H 0.900823 0.648580 3.535955
O 3.527239 -1.298426 0.216606
H 3.448212 -3.263751 0.499449
C 2.839627 -2.342762 0.432245
O 1.617216 -2.456085 0.570787
O -3.290096 1.952325 -0.034974
C -3.699497 2.180930 1.176337
O -4.324547 3.164917 1.525570
H -3.429299 1.396376 1.909802
O -3.393891 -0.858208 0.583587
C -2.832410 -1.853595 1.108703
H -3.495837 -2.500764 1.707132
O -1.637148 -2.209175 1.047552
O -2.352691 -1.059423 -2.069048
C -1.411119 -1.871111 -2.226431
H -1.472902 -2.508079 -3.123735
O -0.408428 -2.057274 -1.501162
O -1.041289 1.423596 -1.596491
C 0.182069 1.307196 -1.402704
H 0.828040 2.054780 -1.885576
O 0.780174 0.430067 -0.714678

Cu3(HCO2)7- Iso. 3
E=-6246.600423
C 3.824484 2.038233 -1.223519
O 3.228277 2.409320 -0.183079
Cu 2.789794 0.362077 0.139312
O 0.642376 0.471724 -0.660628
C 0.121714 1.474596 -1.219582
O -1.090993 1.711935 -1.397313
Cu -2.562999 0.651618 -0.576186
O -2.490201 -0.671254 -2.058687
C -1.655597 -1.575502 -2.288947
O -0.686199 -1.949752 -1.591100
Cu -0.227565 -1.269894 0.194879
O -1.940746 -2.082882 0.959271
C -3.105067 -1.641402 1.042391
O -3.597244 -0.589683 0.561271
O 3.877315 0.821323 -1.547386
O 2.118978 0.503879 1.959323
C 1.048497 0.098993 2.478068
O 0.142067 -0.613970 1.997884
O 3.335583 -1.503357 0.204795
C 2.616945 -2.543704 0.241202
O 1.383505 -2.647322 0.233381
O -3.325685 2.198557 0.263641
C -2.736676 2.702754 1.291771
O -1.723743 2.309048 1.846653
H 4.308509 2.785966 -1.871084
H 0.892736 0.411021 3.522754
H 3.198211 -3.483742 0.283141
H -3.266696 3.603013 1.671104
H -3.813398 -2.263022 1.617998
H -1.790589 -2.120522 -3.238214
H 0.821412 2.232729 -1.600359

Cu3(HCO2)7- Iso. 4
E=-6246.598864
O -4.626861 2.162374 0.219919
C -4.116604 1.955602 1.316363
H -4.589618 2.348071 2.238792
O -3.035206 1.299572 1.536746
Cu -2.396701 0.717462 -0.207052
O -3.379606 -1.000798 -0.012050
C -2.912974 -2.154106 -0.067482
H -3.649157 -2.973633 0.000877
O -1.910751 0.252619 -2.086241
C -1.013312 -0.408697 -2.640390
H -0.975743 -0.344236 -3.740744
O -0.128349 -1.147937 -2.131349
O -1.720249 -2.531134 -0.192232
Cu 0.062450 -1.699289 -0.276998
O -1.197533 2.313091 -0.334224
O 0.402901 0.953389 0.389783
C -0.020564 2.086482 0.004576
H 0.700725 2.912055 -0.035402
O 0.247992 -2.025826 1.645993
C 1.017043 -1.469566 2.464164
O 1.880558 -0.585760 2.263801
H 0.921967 -1.814791 3.506257
O 1.946453 -2.271632 -0.673016
H 3.888528 -2.651917 -0.907035
C 3.141483 -1.930360 -0.536107
O 3.609291 -0.877935 -0.036763
Cu 2.319027 0.467709 0.666341
O 3.016349 2.048811 -0.091155
C 4.160709 2.182075 -0.701088
O 4.634287 3.240650 -1.063053
H 4.695882 1.229418 -0.878655

Cu(HCO2)3-
D1/D0 CI
(EOM-CCSD/6-31+g*)

Cu	0.161464	-0.036921	0.001973
C	2.591377	-0.425152	0.013722
H	3.684235	-0.587035	0.018043
O	1.950654	-0.638987	-1.05612
O	2.032141	-0.03437	1.079746
O	-0.399477	1.468904	-1.021935
C	-0.956386	2.416733	-0.321614
H	-1.242754	3.295841	-0.934325
O	-1.176385	2.414465	0.903645
O	-0.903488	-1.242975	1.024683
C	-1.742584	-1.938372	0.305607
O	-1.896228	-1.884369	-0.93545
H	-2.357728	-2.635808	0.910455

Cu(HCO2)3-
D5/D4 CI
(EOM-CCSD/6-31+g*)

Cu	-0.603181	-0.408212	0.027411
C	-3.039581	0.800836	0.324827
H	-3.72445	1.440938	0.91831
O	-3.427313	0.342275	-0.755812
O	-1.89255	0.631178	0.901179
O	0.639862	-1.539305	-0.818952
C	1.615523	-2.058262	-0.145615
H	2.213041	-2.778654	-0.742707
O	1.918671	-1.855148	1.036057
O	1.222286	1.162487	-0.590571
C	2.428025	1.550356	-0.266557
O	2.768953	2.604329	0.248373
H	3.151673	0.750716	-0.542139

Cu2(HCO2)3-
S1 minimum Isomer 1
(EOM-CCSD/6-31+g*)

C	-0.001181	2.314939	-0.000706
H	-0.001506	3.419868	-0.000872
Cu	-1.107984	-0.249016	0.322039
Cu	1.107344	-0.248463	-0.321984
O	-1.040925	1.756326	0.457786
O	1.038977	1.7568	-0.45884
O	-2.864057	-0.790407	0.997474
O	2.864059	-0.789283	-0.996157
C	-3.602754	-0.850123	-0.057263
C	3.602336	-0.847891	0.058944
O	-3.221738	-0.62831	-1.219942
O	3.22072	-0.625487	1.221307
H	-4.659859	-1.126094	0.130085
H	4.659664	-1.123437	-0.127778

Cu2(HCO2)3-
S1 minimum Isomer 2
(EOM-CCSD/6-31+g*)

Cu	0.000136	1.569482	-0.632668

Cu -0.000147 -1.505168 -0.143627
O 0.00016 1.840751 1.243603
O 1.917915 0.663082 -0.699731
O -1.917813 0.663437 -0.699722
O -0.000013 -0.35606 1.850457
O -1.904888 -1.593658 -0.406203
O 1.904574 -1.594013 -0.406227
C 0.000058 0.863378 2.082263
H 0.000179 1.200754 3.137103
C 2.460651 -0.468001 -0.582435
H 3.564397 -0.490655 -0.649167
C -2.460759 -0.467542 -0.5824
H -3.564506 -0.489998 -0.649173

Cu2(HCO2)3-
T1 minimum Isomer 2
(CCSD/6-31+g*)
cu 1.225907 -1.326283 0.000000
cu -0.327767 0.857446 0.000000
c 0.698519 2.995926 0.000000
h 1.216396 3.968313 0.000000
o 0.405968 2.442741 1.102983
o 0.405968 2.442741 -1.102983
o -1.290803 0.009609 1.443906
o -1.290803 0.009609 -1.443906
c -1.01441 -1.146058 1.923844
c -1.01441 -1.146058 -1.923844
h -1.681702 -1.45622 2.750623
h -1.681702 -1.45622 -2.750623
o -0.109992 -1.932503 1.58037
o -0.109992 -1.932503 -1.58037

Cu2H2-
E=-3282.366014
H -0.825779 -2.570477 -0.000000
Cu -0.002865 -1.262733 -0.000000
Cu -0.002865 1.351192 0.000000
H 0.991967 0.005163 -0.000000

Cu2H-
E=-3281.787708
H -0.000000 -0.000000 -2.716875
Cu 0.000000 -0.000000 -1.165226
Cu -0.000000 0.000000 1.258911

Cu2(HCO2)3H-
E=-3849.602793
C -0.000217 2.548095 0.132300
O -1.129734 2.012175 0.054469
Cu -1.474417 0.088810 -0.195730
O -2.736852 -1.275449 -0.693191
C -3.691416 -1.445373 0.149823
O -3.884797 -0.839556 1.197397
O 1.129641 2.013171 0.052775
Cu 1.474699 0.089570 -0.196654
O 3.883156 -0.841605 1.199518
C 3.691165 -1.445613 0.150666
O 2.737956 -1.273995 -0.693566
H -0.000608 3.641225 0.290977
H 4.397378 -2.247375 -0.156716
H -4.397450 -2.247388 -0.157298
H 0.000379 -0.620081 -0.133777

Cu2(HCO2)4-
E=-4038.278590
Cu -1.634853 0.003560 -0.746066
Cu 0.842941 -0.001026 0.333157
O 0.547685 -2.010485 0.108643
C -0.408692 -2.492981 -0.539374
O -1.389264 -1.903819 -1.059424
O -1.385497 1.911258 -1.051412
C -0.402893 2.496574 -0.530703
O 0.552935 2.009889 0.114892
O -0.030031 -0.002396 2.078378
C -1.283037 -0.006069 2.270828
O -2.195988 -0.006083 1.434764
O 2.103464 -0.000169 -1.159127
C 3.279313 -0.001615 -0.652465
O 3.559386 -0.003507 0.543959
H 4.089244 -0.000896 -1.412015
H -1.564799 -0.009428 3.343022
H -0.380175 3.593689 -0.660045
H -0.388465 -3.589806 -0.671693

Cu2(HCO2)-
E= -3470.484041
Cu -2.172825 -0.467272 0.000000
Cu -0.000000 0.367333 -0.000000
O 1.911984 -0.724691 -0.000000
C 2.947776 -0.017108 -0.000000
O 3.278648 1.192876 -0.000000
H 3.800217 -0.744589 -0.000000

Cu2(HCO2)H-
E= -3471.062961
Cu 1.477863 -1.813670 -0.000000
H 1.492976 -0.105002 -0.000000
Cu 0.000000 0.292686 0.000000
O -1.847084 0.766353 0.000000
C -2.249766 1.983287 0.000000
H -3.362870 2.058077 0.000000
O -1.589110 3.015612 0.000000

Cu3(HCO2)6-
E= -6057.379023
C -3.957931 1.912091 0.215443
Cu -2.582340 -0.032199 -0.079811
O -0.520079 0.877173 0.230791
Cu 0.781434 -0.833479 0.121632
O 2.552156 -1.743121 -0.412050
C 3.532674 -1.178357 -0.944861
O 3.732846 0.048678 -1.127038
Cu 2.474875 1.313432 -0.349717
O -3.792715 1.105358 1.166049
O -3.416825 1.724652 -0.903963
O -2.657054 -1.642669 0.985607
C -1.721139 -2.487235 1.140429
O -0.545997 -2.425234 0.769709
O -2.052983 -0.864833 -1.775616
C -0.929462 -1.122076 -2.275803
O 0.209141 -1.085541 -1.766106
C -0.203217 2.069329 -0.045853
O 0.930104 2.505750 -0.332307
O 1.338987 -0.621053 1.972262
C 2.189720 0.205310 2.421298
O 2.836879 1.067703 1.814015
H -4.587062 2.803148 0.370331
H -2.037798 -3.395661 1.687711
H -0.959857 -1.429305 -3.336524
H -1.024166 2.801255 -0.037869
H 2.349336 0.114758 3.513633
H 4.334929 -1.848366 -1.303036

Cu
E=-1640.542211
cu 0.000000 0.000000 0.000000

Cu(HCO2)
E=-1829.803655
Cu -0.887072 -0.000194 0.000002
O 0.889218 1.110799 0.000033
C 1.483688 0.000155 -0.000163
O 0.890664 -1.110381 0.000034
H 2.583896 0.001353 0.000395

CuH-
E=-1641.144154
H 0.000000 0.000000 -1.550908
Cu 0.000000 -0.000000 0.053480

HCO2
E=-189.149366
c -0.000000 0.000000 0.206679
h -0.000000 0.000000 1.370362

o 0.000000 -1.163677 -0.163152
o 0.000000 1.163677 -0.163152

HOCO
E=-189.162547
C 0.000000 0.409408 0.000000
O 1.166265 0.256116 0.000000
O -0.939853 -0.547192 0.000000
H -1.811304 -0.127832 0.000000

TS(HCO2 → H +CO2)
E=-189.143303
c 0.000000 -0.000000 0.069399
o -0.000000 -1.164859 -0.126895
o 0.000000 1.164859 -0.126895
h 0.000000 -0.000000 1.613922
